# Alternative start and termination sites of transcription drive most transcript isoform differences across human tissues

**DOI:** 10.1093/nar/gkx1165

**Published:** 2017-11-30

**Authors:** Alejandro Reyes, Wolfgang Huber

**Affiliations:** 1 European Molecular Biology Laboratory, Meyerhofstrasse 1, 69117 Heidelberg, Germany; 2 Department of Biostatistics and Computational Biology, Dana-Farber Cancer Institute, Boston, MA, 02215, USA; 3 Department of Biostatistics, Harvard T.H. Chan School of Public Health, Boston, MA, 02215, USA

## Abstract

Most human genes generate multiple transcript isoforms. The differential expression of these isoforms can help specify cell types. Diverse transcript isoforms arise from the use of alternative transcription start sites, polyadenylation sites and splice sites; however, the relative contribution of these processes to isoform diversity in normal human physiology is unclear. To address this question, we investigated cell type-dependent differences in exon usage of over 18 000 protein-coding genes in 23 cell types from 798 samples of the Genotype-Tissue Expression Project. We found that about half of the expressed genes displayed tissue-dependent transcript isoforms. Alternative transcription start and termination sites, rather than alternative splicing, accounted for the majority of tissue-dependent exon usage. We confirmed the widespread tissue-dependent use of alternative transcription start sites in a second, independent dataset, Cap Analysis of Gene Expression data from the FANTOM consortium. Moreover, our results indicate that most tissue-dependent splicing involves untranslated exons and therefore may not increase proteome complexity. Thus, alternative transcription start and termination sites are the principal drivers of transcript isoform diversity across tissues, and may underlie the majority of cell type specific proteomes and functions.

## INTRODUCTION

Alternative splicing, alternative promoter usage and alternative polyadenylation enable the generation of multiple transcript isoforms from a single gene (1–3). In mammalian genomes, at least 70% of genes have multiple polyadenylation sites, >50% of genes have alternative transcription start sites and nearly all genes undergo alternative splicing (4–7). Hence, these molecular processes have the potential to substantially increase the repertoire of transcripts, proteins and functions encoded by mammalian genomes (8–10).

Alternative transcript isoforms regulate important biological processes (11,12), and their mis-expression is associated with diseases, including cancer (13–16). For dozens of genes, alternative transcripts yield alternative proteins with distinct protein interactions, subcellular localization, stability, DNA-binding properties, lipid-binding properties or enzymatic activity (17,18). Recently, it was reported that the majority of alternatively spliced RNAs bind to ribosomes (19), suggesting that they are translated. This finding suggests that the currently known instances of functional protein isoforms could be the tip of an iceberg. However, most alternative exons do not appear to be under selective pressure and show reduced cross-species conservation (20). Furthermore, analyses of protein structures and functional features predict that most alternative transcript isoforms would encode proteins with disrupted structures and functions (21). Indeed, large-scale proteomics surveys indicate that the abundance of isoforms with disrupted domains, if not zero, is generally below levels that can currently be detected with high confidence (22,23). This raises the possibility that the function of a large proportion of transcript isoforms, if any, is on the level of the RNA rather than the protein.

If alternative transcript isoforms function primarily at the mRNA level, one might expect an important role of alternative transcription start and stop sites, since 3′ and 5′ untranslated regions (UTRs) frequently enhance post-transcriptional regulation by fine-tuning the stability and translation of mRNAs (24–27). Alternative transcription start and stop sites have been reported to contribute to isoform diversity more than alternative splicing, based on analyses of transcript annotation databases (28) and of mouse cerebellar development (29).

Previous studies have characterized various aspects of isoform regulation across human tissues. For example, a recent study analyzed 16 RNA-seq samples from the Illumina Body Map and found that for 10–20% of exon-skipping events, splicing ratios differed between any two given tissues (30). Using the same data, another study analyzed the expression of exon–exon junctions and found that 65% of expressed genes contain at least one tissue-specific exon–exon junction (31). By profiling transcriptional cleavage sites, it has been shown that tissue-specific usage of alternative cleavage sites is prevalent (32). While tissue-specific genes tend to have a single transcription cleavage site, genes that are ubiquitously expressed across tissues have multiple cleavage sites, suggesting that the selection of alternative cleavage sites has an important role in the modulation of RNA abundances (24). Similarly, using a protocol to quantitatively assay transcription start sites across 975 human samples, it was shown that the majority of protein-coding genes contain multiple tissue-dependent transcription start sites (4,8). Supplementary Table S1 contains a summary of samples, methods and main findings from recent studies that analyze transcript differences between human tissues. Although these studies characterized tissue-associated differences in either splicing, start sites or cleavage sites, it remains unclear what is the balance of contributions from each of these isoform-generating processes to transcript isoform differences across cell types.

Here, we developed an analytical strategy to approach this question using data from 23 cell types across 94 individuals from the largest collection to date of tissue transcriptomes established by the Genotype-Tissue Expression (*GTEx*) Project V6 (33). We found that there is tissue-specific regulation of alternative transcript isoform choice for a large fraction of the human genome, affecting about half of multi-exonic genes. The majority of these events cannot be explained by alternative splicing; rather, most appear to arise from alternative usage of transcription start and termination sites. Integration of data from the Functional Annotation of The Mammalian Genome (FANTOM) consortium (8) confirmed prevalent tissue-dependent usage of alternative transcription start sites. We also found that although tissue-dependent alternative splicing generates a large diversity of RNA isoforms, most of this diversity is unlikely to be reflected at the proteome level. Furthermore, our results suggest that alternative transcript start and polyadenylation sites play an important role in establishing cell type specificity.

## MATERIALS AND METHODS

### Data processing and sample selection

We downloaded and decrypted the *GTEx* data using the *Short Read Archive Toolkit* software. We used genomic and annotation files of the human reference genome version *GRCh38* as provided by release 84 of *ENSEMBL* (34). To avoid mapping biases, we standardized the read length of all samples. Since most samples consisted of reads of 76 nucleotides (nt), we trimmed the reads to 76 nt for samples with longer reads and excluded samples with shorter read lengths. Next, we mapped the resulting reads to the human reference genome using *STAR v2.4.2a* (35). We provided the aligner with annotated exon–exon junctions and followed the recommended ‘2-pass alignment’ pipeline to optimize mapping accuracy. We excluded samples with <1 000 000 reads mapping uniquely to the reference genome as well as those samples where less that 60% of the reads could be assigned to a unique position in the reference genome. Since the *GTEx* data did not contain the samples for all tissues of each individual, we defined three large subsets of samples that would enable us to analyze each subset as a fully crossed design (containing all tissue-individual combinations) while at the same time keeping as many different individuals and tissues as possible. A description of these subsets, which comprised a total of 798 samples, is given in the ‘Results’ section.

Based on the transcript isoform annotations, we defined reduced gene models with non-overlapping exonic regions (36) using the *HTSeq* (37) python scripts from the *DEXSeq* package. Importantly, reduced gene models enabled us to unambiguously assign reads to exonic regions. For each of the 798 samples, we tabulated the reads to each exonic region. Only reads mapping uniquely to the reference genome were considered for further analysis.

### Relative exon usage coefficients

**Figure 1.**
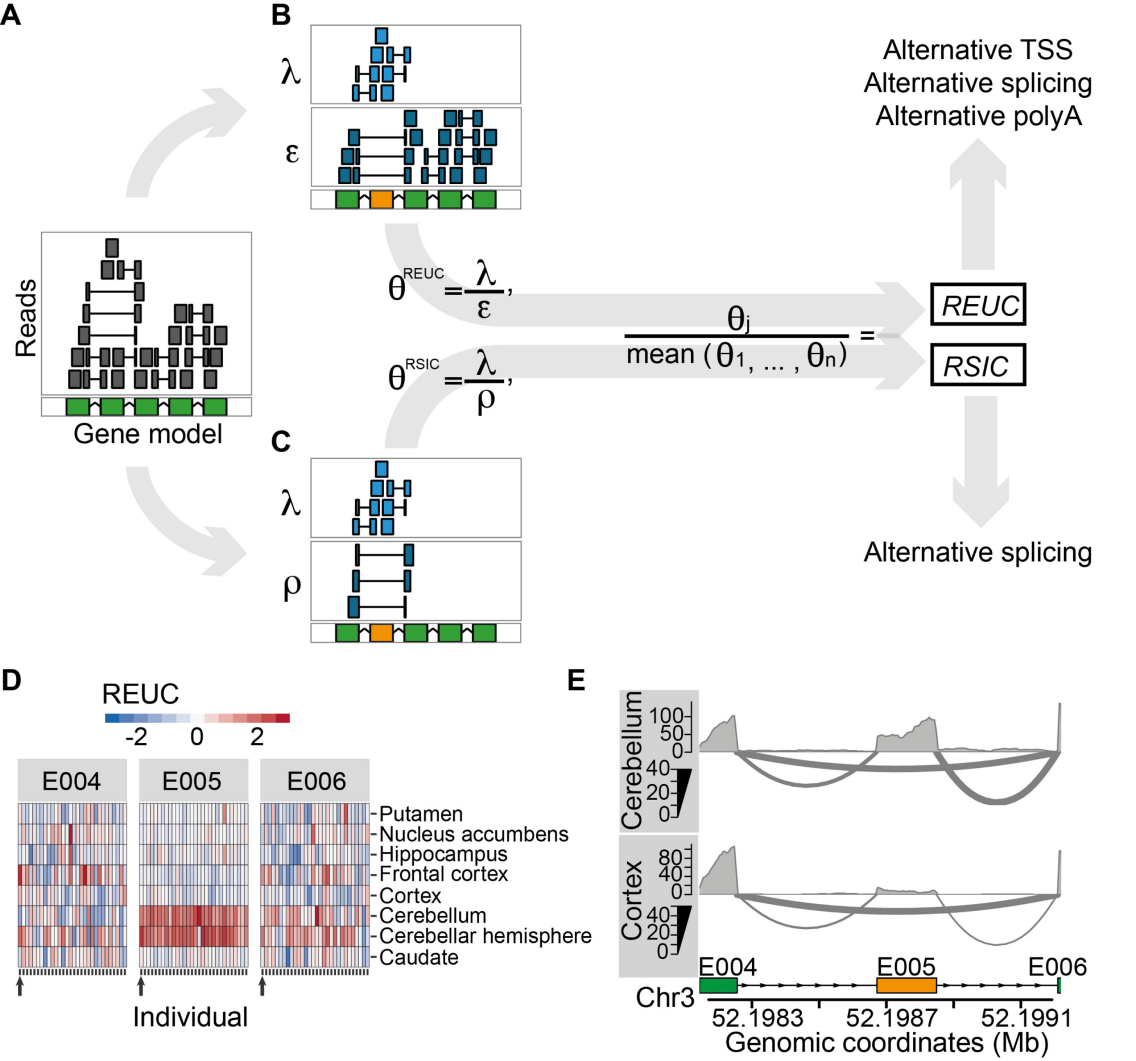
Quantification of exon usage. (**A**) Exemplary gene model in the reference genome (green) and alignments of RNA-seq reads (upper panel). Sequenced fragments whose alignments fall fully into an exonic region are shown by a gray box; alignments that map into two (or more) exonic regions are shown by shorter gray boxes connected by a horizontal line. For a particular exon (highlighted in orange), we consider two strategies to quantify its usage, as illustrated in Panels (**B** and **C**) (see ‘Materials and Methods’ section for the formal description). The first strategy is illustrated in Panel B, where sequenced fragments are counted into two groups: those that map fully or partially to the exon (λ) and those that map to the rest of the exons (ϵ). θ^REUC^ is defined as the ratio between λ and ϵ, and the *REUC* for the exon in sample *j* is computed as the ratio between θ^REUC^ in that sample to the mean θ^REUC^ across all samples. Panel C illustrates the second strategy, where sequenced fragments are also counted into two groups: those that map fully or partially to the exon (λ) and those that align to exons both downstream and upstream of the exon under consideration (ρ). The latter represent transcripts from which the exon was spliced out. θ^RSIC^ is then defined as the ratio between λ and ρ. The relative spliced-in coefficient (*RSIC*) for the exon in sample *j* is the ratio of θ^RSIC^ in this sample to the mean θ^RSIC^ across all samples. Note that while differences in exon usage due to alternative splicing are reflected in both *REUCs* and *RSICs*, differences due to alternative transcription or termination are only reflected in *REUCs*. (**D**) Heatmap representations of the *REUCs* for three exonic regions (*E004, E005* and *E006*) of the gene *5-Aminolevulinate Synthase 1*, computed using s*ubset A* of the *GTEx* data. The rows of the heatmaps correspond to the eight tissues, and each column corresponds to one individual. The horizontal color patterns of exon *E005* indicate elevated inclusion of cerebellum and cerebellar cortex as compared to the rest of the brain cell types. (**E**) RNA-seq samples from two cell types (cortex and cerebellum) from individual *12ZZX* (also indicated by the arrows below each heatmap in Figure 1D) are displayed as sashimi plots. The three exonic regions presented in Panel D are shown. The middle exon, *E005*, is an untranslated cassette exon (ENSEMBL identifier ENSE00002267562) that is spliced out more frequently in cortex than in cerebellum.

We modeled the counts using generalized models of the Gamma-Poisson family for each subset of the *GTEx* data (36,38). We denoted *k*_*ij*1_ as the number of reads mapping to exonic region *i* in sample *j*. When estimating *Relative Exon Usage Coefficients (REUCs), k*_*ij*0_ denoted the sum of reads mapping to exonic regions of the same gene as exonic region *i* but excluding exonic region *i* (Figure 1B). *k*_*ij*0_ and *k*_*ij*1_ are realizations of a random variable *K*_*ijl*_ that is modeled by a Gamma-Poisson distribution,

(1) \begin{equation*} K_{{ijl}} \sim \operatorname{GP}( \text{mean} = s_{{j}}\, \mu _{{ijl}}; \;\text{dispersion} =\alpha _{{il}}), \end{equation*}

where *s*_*j*_ is a scaling factor that accounts for between-sample differences in sequencing depth and α_*il*_ is the dispersion parameter that describes the spread of the count data distribution. *s*_*j*_ is estimated using the *DESeq* method (39) and α_*il*_ is estimated as in *DEXSeq* (36). The mean μ_*ijl*_ was predicted by the model:

(2) \begin{equation*} \log \mu _{ijl} = \beta ^{S}_{ij} + l\beta ^{E}_{i} + lx^{\text{sex}}_{j}\beta ^{\text{sex}}_{i} + l\beta ^{\text{REUC}}_{i,u(j),t(j)}, \end{equation*}

where *l* = 1 when referring to the exonic region *i* and *l* = 0 when referring to the counts from the rest of the exons of the same gene. The coefficients of the model are explained as follows:

- The coefficient $\beta ^{S}_{ij}$ represents overall gene expression effects on sample *j*.
- Since $\beta ^{E}_{i}$ is only included when *l* = 1, it estimates the mean across samples of the logarithmic ratio between the counts from exon *i* with respect to the counts of the rest of the exons of the same gene (i.e. *K*_*ij*1_/*K*_*ij*0_). Therefore, this coefficient is a measure of the average exon usage across all samples.
- The coefficient $\beta ^{\text{sex}}_{i}$ captures sex-dependent differences in exon usage. Including it in the model prevents confounding in situations of unbalanced sex distribution among the individuals, and reduces noise otherwise. In the Generalized Linear Model (GLM) model matrix, $x^{sex}_{j}$ takes the value of −1/2 if sample *j* is from a male individual and 1/2 if sample *j* is from a female individual. Thus, this coefficient estimates the logarithmic fold change of the usage of exonic region *i* for each sex with respect to the average exon usage.
- The *REUC*, $\beta ^{\text{REUC}}_{i,u{(j)},t{(j)}}$, is the interaction coefficient between individual $u{(j)}$ and tissue $t{(j)}$ from which sample *j* was taken. For exonic region *i*, the coefficient $\beta ^{\text{REUC}}_{i,u{(j)},t{(j)}}$ thus estimates the logarithmic fold change in exon usage for each individual-tissue combination with respect to the average exon usage.

The *REUCs* are subjected to an empirical Bayes shrinkage procedure in order to improve their precision (38,40).

### Relative spliced-in coefficients

To estimate *relative spliced-in coefficients* (*RSICs*), we used Equations (1) and (2) to model a modified read counting scheme. *k*_*ij*1_ remains the same as for the *REUCs* fit but *k*_*ij*0_ (i.e. *l* = 0) now denotes the number of reads supporting the splicing out from transcripts of exonic region *i* (Figure 1C). For exonic region *i*, the coefficient $\beta ^{E}_{i}$ from Equation (2) now measures the mean across samples of the logarithmic ratio between the number of reads supporting the splice in of exonic region *i* and the number of reads supporting the splice out of exonic region *i* (i.e. the average spliced-in (*SI*) coefficient). The coefficient $\beta ^{\text{sex}}_{i}$ for exonic region *i* now measures the change of *SI* between each sex with respect to the average *SI*. The *RSIC* for exon *i*, $\beta ^{\text{RSIC}}_{i,u{(j)},t{(j)}}$, measures the logarithmic fold change in the exon’s *SI* for each tissue-individual combination with respect to the average *SI*. As for the *REUCs*, the *RSICs* are also subjected to the empirical Bayes shrinkage procedure to eliminate the mean-variance trend (38).

Changes in exon usage driven by alternative splicing are reflected in both *REUCs* and *RSICs*. Changes in exon usage due to alternative initiation or termination sites of transcription, which do not result in exon–exon junction reads, are only reflected by *RSICs*.

### Estimation of tissue-dependance score

For each exonic region on each subset of the data, we estimated a score based on the *REUCs* to measure to what extent the usage of each exonic region was tissue-dependent. First, the *REUCs* of a given exonic region *i* were expressed as the number of standard deviations away from the median of the exon’s *REUCs*,

(3) \begin{equation*} Z_{iut} = \frac{\beta ^{\text{REUC}}_{iut} - \displaystyle {median}_{u,t} (\beta ^{\text{REUC}}_{iut})}{\displaystyle {\sigma }_{u,t} ( \beta ^{\text{REUC}}_{iut} )}. \end{equation*}

Then, the tissue-dependence score for exonic region *i* was defined by:

(4) \begin{equation*} T_{i} = \displaystyle {max}_{t}\bigg \lbrace \left|\frac{1}{m}\displaystyle \sum _{u=1}^{m} Z_{iut}\right|\bigg \rbrace , \end{equation*}

with *m* being the number of individuals on the data subset.

### Analysis of variance of *REUCs* and *RSICs*

For each exonic region on each subset of the data, we fitted an analysis of variance model,

(5) \begin{equation*} \beta ^{\text{REUC}}_{iut} = \beta ^{0}_{i} + \beta ^{\text{Individual}}_{iu} + \beta ^{\text{Tissue}}_{it} + \epsilon _{iut}, \end{equation*}

using ordinary least squares regression to minimize the residual sum of squares (RSS),

(6) \begin{equation*} \text{RSS}_{i} = \sum _{u,t} \epsilon ^{2}_{iut} = \sum _{u,t} ( \beta ^{\text{REUC}}_{iut} - \hat{\beta }^{\text{REUC}}_{iut} )^{2}, \end{equation*}

where $\hat{\beta }^{{REUC}}_{iut}$ are the *REUC* values predicted by the model. In order to estimate the coefficient of partial determination (*R*^2^) for the tissue predictor (i.e. the proportion of total variance that can be attributed to variance across tissues), we fitted a reduced model lacking the $\beta ^{\text{Tissue}}_{it}$ term,

(7) \begin{equation*} \beta ^{\text{REUC}}_{iut} = \beta ^{0}_{i} + \beta ^{\text{Individual}}_{iu} + \epsilon _{iut}. \end{equation*}

The *R*^2^ for a given exon *i* was then calculated by,

(8) \begin{equation*} R^{2}_{i} = 1 - \frac{\text{RSS}_{i}(\text{full})}{\text{RSS}_{i}(\text{reduced})}, \end{equation*}

where, RSS_*i*_(full) is the RSS from the full model (i.e. Equation (5)) and RSS_*i*_(reduced) is the RSS from the reduced model (i.e. Equation (7)). The same procedure was followed to estimate *R*^2^ on the *RSICs* but using $\beta ^{\text{RSIC}}_{iut}$ as the response variable in Equation (5) and in Equation (7).

### Genomic analyses

To test for over-representation of features among the genes with tissue-dependent usage (TDU), we used the *R CRAN* package *MatchIt* (41) to generate background sets of genes with the same distribution of expression strength and number of exonic regions as the genes with TDU. Genes were classified according to *ENSEMBL* annotations and we used a χ^2^-test for differences between genes with TDU and the background set of genes. Gene biotypes were retrieved from *ENSEMBL* using the *Bioconductor* (42) package *biomaRt* (43). For enrichment of features among exons with TDU, we also used *MatchIt* to generate background sets of exons with the same distribution of expression strength and exon widths. We tested for differences between exons with TDU and the background set of exons using a χ^2^-test.

Operations on genomic ranges were done using the *Bioconductor* package *GenomicRanges* (44). Data visualizations and graphics were generated using the *Bioconductor* packages *ggplot2* (45) and *Gviz*(46).

## RESULTS

### Quantitative analysis of transcript isoform regulation across tissues.

To evaluate the scope and regulation of differential transcript isoforms in humans, we analyzed transcriptome data (RNA-seq) from the V6 release of the *GTEx* project (33). The overall dataset comprises 9795 RNA-seq samples from 54 tissues from a total of 551 human individuals. Since the dataset does not contain each tissue for each individual, we identified subsets of data that could be analyzed as fully crossed designs (i.e. contained all possible tissue-individual combinations). We mapped the sequenced fragments to the human reference genome version GRCh38, obtained from ENSEMBL release 84 (34), using the aligner *STAR v2.4.2a* (35). We excluded samples where the number of reads mapping uniquely to the reference genome was below 1 000 000 or where the percentage of uniquely mapping reads was below 60%. Using these data quality criteria, we defined three subsets of *GTEx* data for our analyses. Subset A consisted of eight brain cell types (frontal cortex [BA9], nucleus accumbens, putamen, cortex, cerebellum, caudate, cerebellar hemisphere and hippocampus) across 30 individuals. Subset B included nine tissues (skeletal muscle, thyroid, whole blood, lung, subcutaneous adipose, skin, tibial artery, tibial nerve and esophagus [mucosa]) from 34 individuals. Subset C comprised six tissues (heart, aorta, esophagus [muscularis], pancreas, colon and stomach) from 42 individuals. These subsets were non-overlapping, and altogether our analysis employed 798 unique samples from the *GTEx* dataset.

For each gene, we determined its non-overlapping exonic regions (36) based on the *ENSEMBL* transcript annotations (‘Materials and Methods’ section). We obtained 499 667 non-overlapping exonic regions in 35 048 multi-exonic genes, of which 412 116 belonged to 18 295 protein-coding genes. For each subset, we computed two measures of exon usage per exonic region: *REUCs* (38) and *RSICs*. Both coefficients measure exon usage in a specific tissue in a particular individual relative to the average exon usage across all tissues and individuals (‘Materials and Methods’ section). The *REUC* defines exon usage as the fraction of sequenced fragments that map to the exonic region among all fragments mapping to the rest of the exonic regions from the same gene. In contrast, the *RSIC* measures the fraction of sequenced fragments that map to the exonic region compared to the number of reads that support the skipping of that exonic region via alternative splicing (Figure 1C). Note that differences in exon usage due to alternative splicing are reflected in both *REUCs* and *RSICs* (Figure 1B and C). Changes in exon usage due to alternative transcription initiation sites or alternative polyadenylation sites, which do not result in exon–exon junction reads, are only reflected in *REUCs* (Figure 1D).

We exemplify the analysis on the *5-Aminolevulinate Synthase 1* (*ALAS1*) gene (Figure 1D and E). *ALAS1* encodes an enzyme required for the biosynthesis of heme, a co-factor essential for the proper function and differentiation of many cell types, including those of the hematopoietic, hepatic and nervous systems (47). Induction of *ALAS1* has been associated with acute attacks of porphyria disease (48). By exploring the *REUCs* for *ALAS1*, we found that a 5′ untranslated exon was included more frequently in the transcripts generated in cerebellum and cerebellar hemisphere than in the other brain cell types (E005, Figure 1D). The same pattern of TDU was also evident from the *RSICs* (Supplementary Figure S1), which indicates that the TDU pattern is a consequence of alternative splicing rather than alternative transcription initiation or termination (Figure 1E). *ALAS1* transcripts that include this 5′ exon are resistant to heme-mediated decay, and their translation is inhibited in cultured cells (49). The detected splicing pattern suggests that *ALAS1* is post-transcriptionally regulated differently in cerebellum than in the rest of the brain.

To further validate our quantitative approach on the *GTEx* data, we compared our results to a series of tissue-dependent splicing events that were previously characterized based on different data, different experimental assays and/or different computational methods. We show ten such cases in the Supplementary Material, involving the genes *SLC25A3* (6) (Supplementary Figure S2), *MEF2C* (50) (Supplementary Figure S3), *ANK3* (51) (Supplementary Figure S4), *SGCE* (52) (Supplementary Figure S5), *MYO1C* (53) (Supplementary Figure S6), *KSR1* (54) (Supplementary Figure S7), *ATP11B* (55) (Supplementary Figure S8), *TPD52* (55) (Supplementary Figure S9), *ATP5C1* (56) (Supplementary Figure S10) and *NDUFV3* (57) (Supplementary Figure S11). These examples demonstrate how *REUCs* and *RSICs* capture tissue-dependent patterns of exon usage that had been previously characterized using different experimental and computational approaches.

### Tissue-dependent usage of exons is widespread in humans.

**Figure 2.**
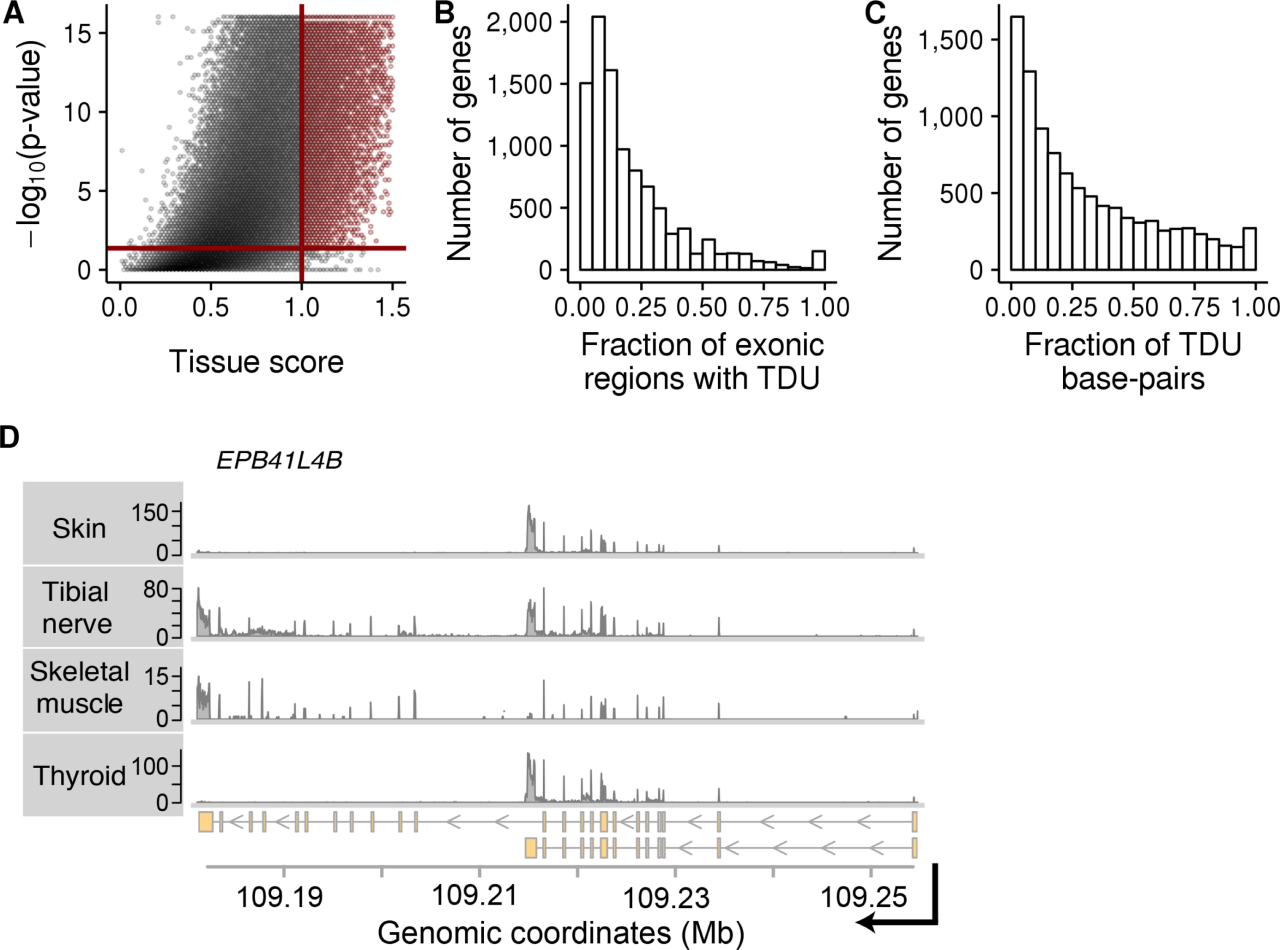
Tissue-dependent exon usage is widespread in the human genome. Panels (**A**–**C**) show data from *subset A* of the *GTEx* data. The same plots using data from subsets *B* and *C* can be found in Supplementary Figures S12 and S14. (A) Similar to a volcano-plot, this figure shows statistical significance (*P*-value on −log_10_ scale) versus effect size (tissue score) of our tissue-dependence test for each exonic region of the human genome. The solid red lines show the thresholds used in this study to call an exonic region tissue-dependent. The *P*-value threshold 4.28 · 10^−2^ corresponds to an *adjusted P-value* of 0.1 according to the Benjamini–Hochberg method to control FDR. (B) Histogram of the fraction of exonic regions within each gene that are subject to TDU (*X*-axis). The *Y*-axis shows the number of genes. (C) Similar to Panel B, but expressed in terms of fraction of base-pairs within a gene affected by TDU. (**D**) Exemplary data from four out of nine tissues of individual *131XE* from subset B. Shown is RNA-seq coverage (*Y*-axis) plots along genomic coordinates (*X*-axis) at the locus of the gene *EPB41L4B* on chromosome 9. The lower panel shows the transcript annotations for this gene. Skin and thyroid express short isoforms, while tibial nerve and skeletal muscle express longer isoforms.

We observed multiple instances of tissue-dependent exon usage analogous to that for the *ALAS1* gene. To investigate how widespread this phenomenon is across the human genome, we defined a tissue score based on the *REUCs* that measures the strength of TDU of an exonic region (‘Materials and Methods’ section). Based on this, we considered an exonic region to be tissue-dependent if its differential usage pattern was statistically significant at a false discovery rate (FDR) of 10%, according to the *DEXSeq* method (36), and if it had a tissue score >1. We found that 23% of the exonic regions (116 601 out of 499 667; Supplementary Figure S13) and 43% of the genes displayed TDU in at least one of the three *GTEx* subsets. Specifically, TDU was observed for 9% (47 659/499 667) of exonic regions and 28% (9839/35 048) genes in subset A, 15% (76 562/499 667) of exonic regions and 35% (12 295/35 048) of genes in subset B, and 6% (30 719/499 667) of exonic regions and 20% (7025/35 048) of genes in subset C (Figure 2A and Supplementary Figure S12). For highly expressed genes, defined as those with an average of at least 100 sequenced fragments, these fractions were even larger (Supplementary Table S2). For example, 65% of highly expressed genes within subset A (8741/13 535) showed differential usage of at least one exonic region. Furthermore, the set of genes with TDU was enriched for protein-coding genes compared to a background set of genes matched for expression strength and number of exonic regions (*P*-value < 2.2 · 10^−16^, odds-ratio = 3.4; Supplementary Table S3), suggesting that TDU plays a substantial role in regulating the tissue specificity of the proteome.

We next investigated the nature of transcript isoform differences between tissues. For each gene containing exons with TDU, we estimated the fraction of exonic regions that were subject to TDU and the fraction of altered exonic nucleotides. For most tissue-dependent genes, a relatively small fraction of exons displayed TDU (Figure 2B, Figure 2C and Supplementary Figure S14). For instance, <25% of exonic regions were differentially regulated in 70% of the subset A genes with TDU. Further, <25% of nucleotides were affected in 53% of the subset A genes with TDU (Supplementary Table S4). The remaining cases, where a larger fraction of the gene displayed tissue-dependent regulation, reflected the expression of substantially different, tissue-specific isoforms. For example, all 27 exonic regions of the gene *Erythrocyte Membrane Protein Band 4.1 Like 4B* (*EPB41L4B*) showed similar expression in tibial nerve and skeletal muscle, which was distinct from the other tissues in subset B of the *GTEx* (Supplementary Figures S15 and S16). This pattern can be explained by the two annotated transcript isoforms of the gene: whereas most cell types in *subset B* tend to express the short isoform, tibial nerve and skeletal muscle preferentially express the longer isoform (Figure 2D).

Thus, transcript isoform regulation across tissues is pervasive across the human genome, particularly for protein-coding genes. In general, a small proportion of exons and nucleotides of genes are changed in tissue-dependent isoforms.

### Alternative transcriptional initiation and termination drive most transcript isoform differences between tissues.

**Figure 3.**
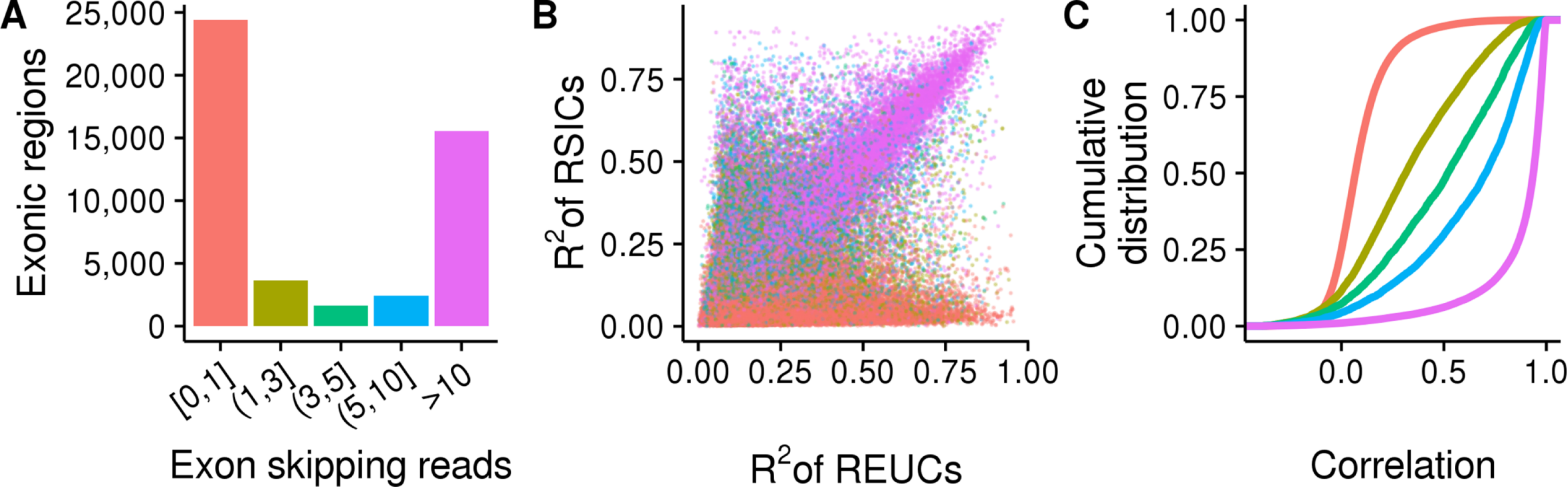
Alternative splicing underlies only a minor fraction of exons with TDU, while the rest are consistent with alternative transcription start or stop sites. The three panels show data from *subset A* of the *GTEx* data. Analogous plots for subsets *B* and *C* are shown in Supplementary Figure S17. (**A**) The heights of the bars show the number of exonic regions with TDU, grouped according to the number of reads that support their splicing out from transcripts. Most exonic regions with TDU have either no or weak evidence of being spliced out from transcripts (bar colored in pink salmon). The bar colors serve also as color legends for Figure 3B and C. (**B**) Each point represents one of the 47 659 exonic regions that were detected to be used in a tissue-dependent manner. The *X*-axis shows the fraction of *REUC* variance that is attributed to variance between tissues (*R*^2^). Analogously, the *Y*-axis shows the *R*^2^ statistic for the *RSICs*. Exonic regions with strong evidence of being spliced out from transcripts (purple points) lay along the diagonal. (**C**) Cumulative distribution functions of the Pearson correlation coefficients between the REUCs and the RSICs are shown for exonic regions with TDU. The regions are stratified according to the number of sequenced fragments supporting their splicing out from transcripts. The *REUCs* and *RISCs* are highly correlated for the minor fraction of exons that have strong evidence of being spliced out from transcripts (purple line).

The example of *EPB41L4B* shows tissue-dependent expression of transcript isoforms that is driven not by alternative splicing, but by the usage of an alternative polyadenylation site, here also referred to as transcription termination site (Figure 2D). Therefore, we asked what fraction of exon TDU is driven by alternative splicing versus alternative transcription start or termination sites. For each exonic region, we searched for evidence of alternative splicing by counting the number of sequenced fragments that supported exon skipping in each sample (Figure 1C). We found that a minor fraction of exonic regions with TDU had appreciable evidence of being spliced out from transcripts (Supplementary Table S5). For instance, the mean of read counts supporting exon skipping was larger than 10 in only 30% (9282) of the exonic regions with TDU in subset C. On the other hand, 53% (16 385) showed no or only weak evidence of being alternatively spliced (Figure 3A and Supplementary Figure S17). We estimated that alternative splicing explains tissue-dependent transcript differences for, at most, 35% of the genes (Supplementary Table S6).

As a second line of evidence, we quantitatively compared the relative exon usage and SI coefficients (*REUCs* and *RSICs*, as defined above). For each exonic region and each subset of the *GTEx* data, we fit two analysis-of-variance models, one for the *REUCs* and one for the *RSICs*, using tissues and individuals as categorical covariates. We determined the coefficient of partial determination (*R*^2^) of the tissue covariate for each fit. A large value of *R*^2^ in the *RSIC* fit indicates that the TDU arises only from alternative splicing. Conversely, a large *R*^2^ in the *REUC* fit indicates that the TDU arises from alternative splicing, alternative transcription initiation sites or alternative transcriptional termination sites. The *REUCs* and the *RSICs* were highly correlated for the minority of exonic regions with TDU that also had strong evidence of alternative splicing, and their *R*^2^ statistics were in good agreement, confirming that the TDU was due to alternative splicing (Figure 3B and C; Supplementary Figure S17). Nevertheless, for the majority of exonic regions with TDU, the TDU was consistent with alternative transcription initiation and termination sites.

### Analysis of CAGE data confirms prevalent tissue-dependent usage of alternative transcription start sites.

To further investigate the hypothesis that alternative start sites substantially drive transcript isoform diversity across tissues, we analyzed the Cap Analysis of Gene Expression (CAGE) data from the *FANTOM* consortium (8). These data provide genome-wide quantitative information of transcriptional start sites (TSS) for many cell types. For each subset of the *GTEx* data, we generated a subset of *FANTOM* samples with the same composition of cell types (as long as the samples existed and had replicates). For instance, based on the cell types from subset A of the *GTEx* data, we selected a set of *FANTOM* samples consisting of caudates, cerebellums, cortexes, hippocampus and putamens. Then, for each of the three subsets of the *FANTOM* data, we tested each gene for changes in the relative usage of alternative TSS across cell types. At a false discovery rate of 10%, we found 2402, 6763 and 2778 genes with TDU of TSS across subsets A, B and C, respectively. Furthermore, the three lists of genes with differential TSS usage were in very good agreement with the counterpart lists of genes with TDU from the *GTEx* subsets (Supplementary Table S7). When considering the genes with differential TSS usage across cell types, 79% (1904) of *subset A*, 80% (5427) of *subset B* and 60% (1657) of *subset C* also showed transcript isoform regulation in the corresponding *GTEx* subsets.

**Figure 4.**
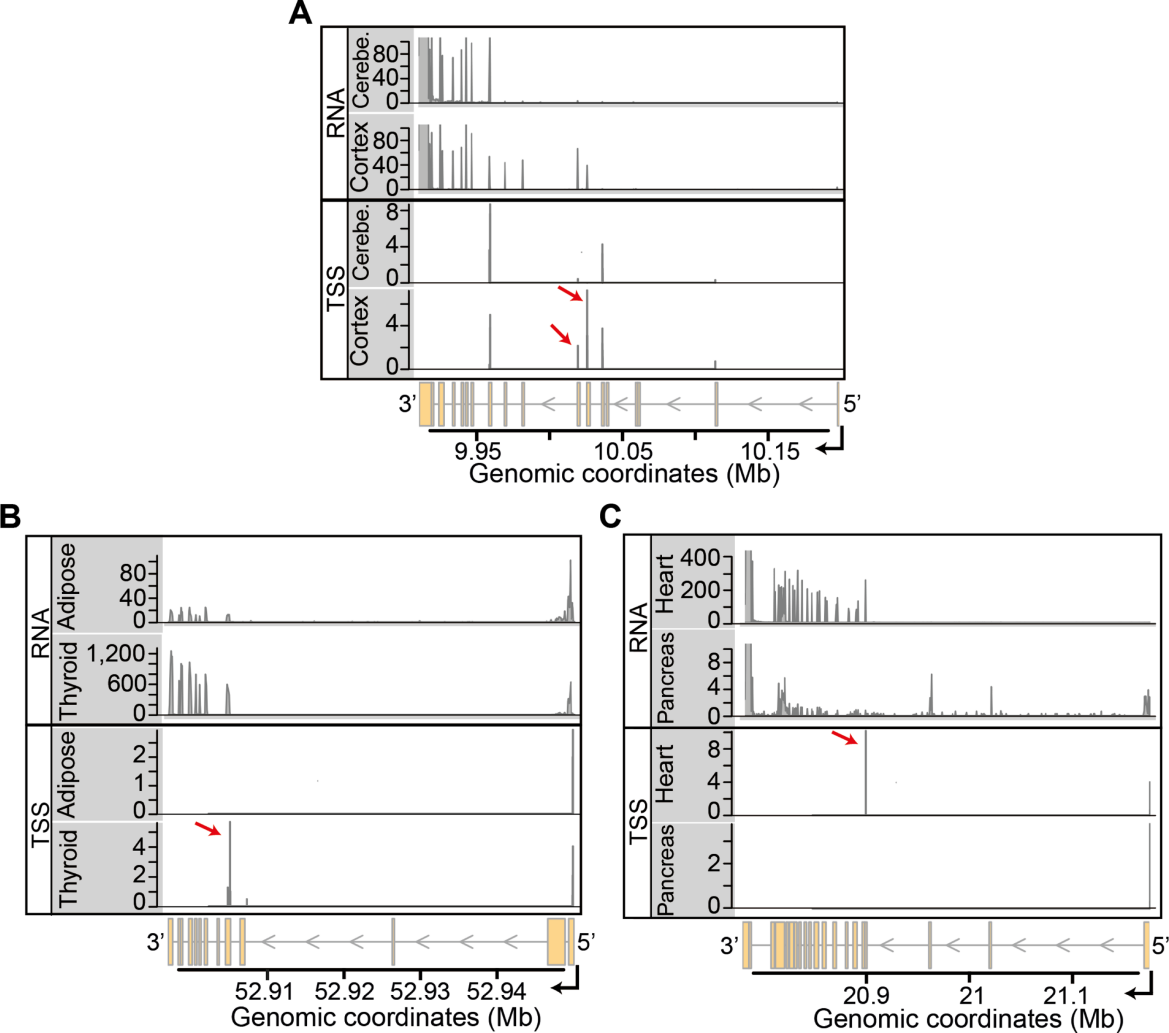
Integration of RNA-seq and CAGE data. Each panel displays an example of a gene where the usage of alternative transcription start sites explains the patterns of TDU. (**A**) Coverage tracks (*Y*-axes) of RNA-seq and CAGE data for cerebral cortex and cerebellum are shown along the genomic coordinates (*X*-axis) of the locus of gene *GAS7*, located on chromosome 17. The upper two tracks show RNA-seq data from individual *12ZZX*. The lower two tracks show mean CAGE counts (on log_2_ scale) for each annotated TSS. Cortex uses two transcription start site clusters (see red arrows) that are absent in cerebellum. The differential usage of these two TSS explains the upstream RNA-seq coverage seen in cortex. (**B**) Analogous to Figure 4A, showing data of thyroid and subcutaneous adipose tissue along the genomic coordinates of the *KRT8* locus on chromosome 12. The RNA-seq data are from individual *11EI6*. The internal TSS cluster that is indicated by the red arrow is strongly used in thyroid tissue, resulting in the expression of short transcript isoforms. (**C**) Same as in Figure 4A, but showing data of heart and pancreas along the genomic coordinates of the *NEBL* locus on chromosome 10. The RNA-seq data corresponds to the individual *ZF29*. In heart, the usage of an internal TSS (indicated by the red arrow) results in the expression of transcript isoforms that exclude several 5′ exons of the gene.

Figure 4 shows three examples of genes with tissue-dependent exon usage patterns that were explained by the usage of an alternative TSS. The first example, from subset A, is *Growth Arrest Specific 7* (*GAS7*, Supplementary Figure S18). From the coverage of sequenced RNA fragments along the genome, we suspected that transcription initiated more downstream in cerebellum as compared to cerebral cortex. The CAGE data revealed five major clusters of TSS for *GAS7*, of which two were strongly used in cerebral cortex and were practically absent from cerebellum (Figure 4A). The differential usage of these two TSS clusters explained the upstream transcription seen in cerebral cortex that was not observed in cerebellum. Similarly, by exploring the data for the gene *Keratin 8* (*KRT8*) in *subset B*, we found patterns of TDU that were very prominent in thyroid tissue compared to subcutaneous adipose tissue (Supplementary Figure S19). These patterns of TDU were explained by the usage of a TSS located in the middle of the gene body that resulted in the expression of shorter transcript isoforms. This internal TSS of *KRT8* was used very frequently in thyroid tissue and was absent in subcutaneous adipose tissue (Figure 4B). We found the exact same pattern for the gene *Nebulette* (*NEBL*) in *subset C* of the data. For this gene, the usage of an internal TSS resulted in transcript isoforms that excluded several 5′ exons. This internal TSS was used very frequently in heart tissue, whereas it was absent in pancreas tissue (Figure 4C and Supplementary Figure S20).

Our integrative analysis of two orthogonal sources of data (independent samples, different technologies) confirms that there is an abundance of alternative TSSs that are used in a tissue-dependent manner and that result in TDU.

### Tissue-dependent splicing of protein-coding exons is rare

Next, we asked which regions of genes were subject to tissue-dependent exon usage. We integrated information from the ENSEMBL and APPRIS databases to annotate each exonic region. Importantly, APPRIS uses information about protein structures, functional data, selective pressure analyses and cross-species conservation to infer which transcript isoforms are likely to encode functional proteins and flags these as principal isoforms, whereas the rest of the transcripts are marked as non-principal isoforms (21).

Using these sources of information, we classified each exonic region into five categories: (i) exonic regions encoding principal isoforms, (ii) exonic regions encoding only non-principal isoforms, (iii) 5′ untranslated exonic regions (5′ UTR), (iv) 3′ untranslated exonic regions (3′ UTR) and (v) untranslated exons belonging to non-coding processed transcripts. Then, for each subset of the *GTEx* data, we generated a background set of exons with the same distributions of mean counts and exon widths.

**Figure 5.**
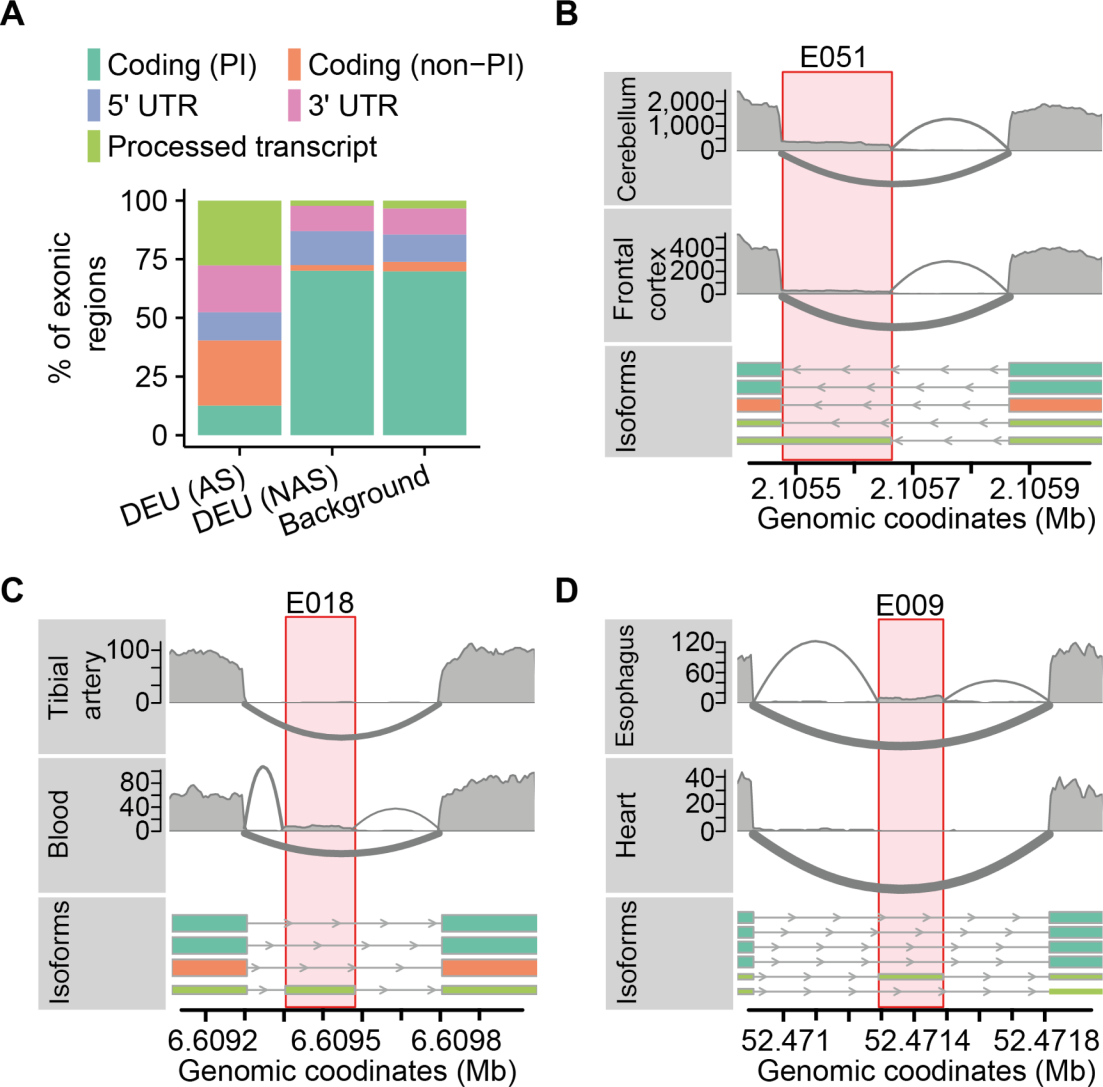
Alternative splicing is infrequent among coding exons. (**A**) The percentage of exonic regions (*Y*-axis) is shown for three subsets of exons: (i) exonic regions with TDU due to alternative splicing [DEU (AS)], (ii) exonic regions with TDU without evidence of alternative splicing [DEU (NAS)] and (iii) a background set of exons matched for expression and exon width. Each color represents a different category of exons according to transcript biotypes: exons coding for principal transcript isoforms [Coding (PI)], exons coding for non-principal transcript isoforms [Coding (non-PI)], 5′ UTRs, 3′ UTRs and exons from non-coding processed transcripts [Processed transcripts]. (**B**) Sashimi plot representation of the RNA-seq data from frontal cortex and cerebellum of individual *WL46*. The lower data track shows the transcript isoforms of the gene *PKD1*. The transcripts are colored according to their biotype (the color legend is the same as in Figure 5A). The highlighted exon (*E051*) belongs to a non-coding transcript and is differentially spliced across tissues. (**C**) Same as in Figure 5B, but showing data from tibial artery and whole blood of the individual *ZTPG*. Transcripts from the gene *MAN2B2* along chromosome 4 are shown. The highlighted exon (*E018*) belongs to a non-coding transcript and is differentially spliced across tissues. (**D**) Same as in Figure 5B, but showing data from esophagus tissue (muscularis) and heart tissue (left ventricle) of the individual *111YS*. The lower track shows the transcripts annotated for gene *NISCH* along chromosome 3. The highlighted exon (*E009*) belongs to a non-coding transcript and is differentially spliced across tissues.

We found that the proportions among the five exon categories were different between exonic regions with TDU arising from alternative splicing (TDU-AS), exonic regions with TDU but no evidence of alternative splicing (TDU-NAS) and the background sets of exons (*P*-value < 2.2 · 10^−16^, χ^2^-test; Figure 5A, Supplementary Figure S21 and Table S8). Exonic regions with TDU-AS were depleted among those coding for principal isoforms and enriched among exonic regions coding for non-principal isoforms and 3′ UTRs. Our analysis also revealed that exons from non-coding processed transcripts, despite being weakly expressed, were alternatively spliced very frequently in a tissue-dependent manner (Figure 5A–C; Supplementary Figures S21 and S22).

Exonic regions with TDU-NAS showed a slight yet significant enrichment among 5′ UTR exons compared to the background (*P*-value < 1.2 · 10^−7^, χ^2^-test; Figure 5A and Supplementary Figure S21). It also occurred frequently among 3′ UTR regions compared to the background (*P*-value < 1.2 · 10^−7^, χ^2^-test), however, we observed this only in *subsets B* and *C* of the *GTEx* data (Supplementary Figure S21).

## DISCUSSION

We analyzed transcript isoform diversity across 798 human transcriptomes covering 23 different cell types. This large and comprehensive dataset together with the analytical approach illustrated in Figure 1A–C enabled us to identify alternative transcription initiation and polyadenylation sites as the principal sources of transcript isoform differences across human cell types. It has been suggested that the regulation of gene expression levels is the main driver of cell type specificity, with splicing playing a complementary role (58). Our analysis suggests that alternative transcription initiation and polyadenylation sites make a sizeable contribution to cellular phenotypes in normal human physiology, and that this contribution is more prevalent than that of splicing. Our analysis highlights two important aspects of RNA splicing. First, alternative splicing is not the main process by which transcript isoform diversity is regulated across tissues. Second, most of the splicing that is regulated differentially across tissues affects untranslated transcripts or non-principal isoforms, and therefore may not have direct consequences on proteome isoform diversity.

Transcriptome-wide studies have shown that most genes express one major isoform at high levels in a given cell type, whereas the remaining (‘minor’) isoforms are expressed at lower levels (9,59). Importantly, protein isoforms detected in large-scale proteomic experiments are consistent with both the major RNA isoforms and the principal isoforms from the APPRIS database (20). We found that tissue-dependent splicing is enriched among untranslated exons, particularly among exons from non-coding transcript isoforms. Further, tissue-specific splicing is depleted among exons encoding principal protein isoforms. Indeed, the exon categories that display abundant tissue-dependent splicing are weakly expressed. Thus, many patterns of tissue-specific splicing could be explained by tissue-specific expression of minor transcript isoforms. Together, these results suggest that most tissue-dependent splicing does not contribute to proteomic isoform diversity. Only around 15% of tissue-dependent splicing involves exons from principal isoforms. Although these splicing events are only a minority, they could result in different protein isoforms if they are translated.

The remaining open question is, if tissue-dependent splicing has little effects at the proteome level, what are its functions at the transcriptome level? Since patterns of splicing of untranslated exons are very frequently tissue-dependent, it seems unlikely that these splicing events are all just noise. A parsimonious possibility is that tissue-dependent splicing plays a widespread role in post-transcriptional regulation, as in the example of the gene *ALAS1*(47). Recent CRISPR-mediated interference screens identified 499 long non-coding RNAs that were essential for cell growth, of which 89% of these showed growth-modifying phenotypes that were exclusive to one cell type (60). Similar screens at the transcript isoform level would be instrumental to evaluate the essentiality of the thousands of non-coding and non-translated tissue-specific isoforms derived from protein-coding loci.

Alternative usage of promoters, splice sites and polyadenylation sites are highly interleaved (1,61). While alternative splicing may have limited effects on protein complexity in normal human physiology, it remains to be seen to what extent tissue-dependent choice of alternative start and termination sites results in truncated versions of proteins. Furthermore, it will be important to investigate to what extent misregulated splicing results in protein isoforms that contribute to disease phenotypes.

## DATA AVAILABILITY

The raw data used for this project are part of the *GTEx* Project and can be found in dbGAP under the accession identifier phs000424.v6.p1. The package *HumanTissuesDEU* contains the data and code needed to reproduce the analysis and figures presented in this manuscript (https://github.com/areyesq89/HumanTissuesDEU).

## Supplementary Material

Supplementary Data
