# Supplementary material for: Alternative start and termination sites of transcription drive most transcript isoform differences across human tissues
Source: Nucleic Acids Res. 2017 Nov 30;46(2):582–92. doi: 10.1093/nar/gkx1165 (PMC5778607; doi:10.1093/nar/gkx1165)
Supplement: Supplementary Data [file gkx1165_supp.pdf]

Supplementary Information: Alternative start and termination sites of transcription drive most transcript isoform differences across human tissues

Alejandro Reyes and Wolfgang Huber

1 Supplementary Figure:

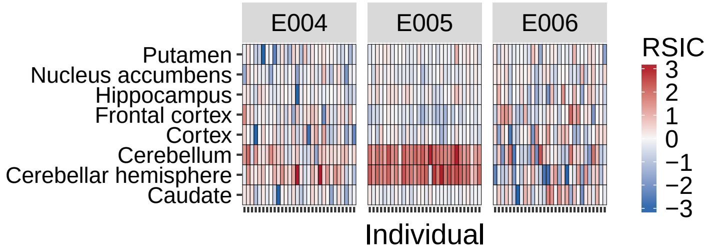

Figure S1: Heatmap representation of relative spliced-in coefficients (*RSICs*) for three exonic regions (*E004*, *E005* and *E006*) of the gene *ALAS1* on *subset A* of the *GTEx* data. Each row of the heatmap shows data for one tissue and each column shows data for one individual. Colors represent the *RSIC* values.

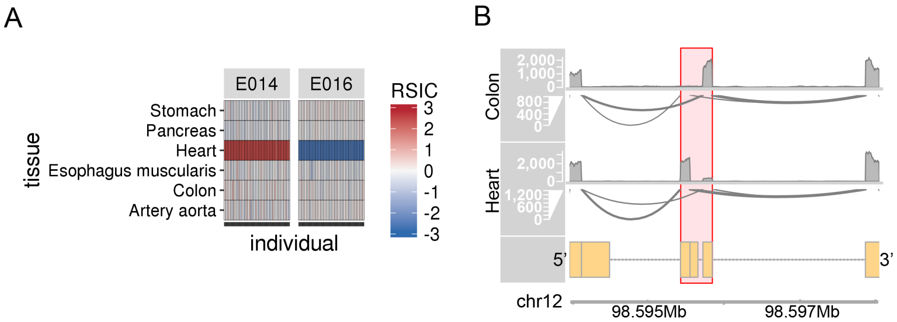

Figure S2: Panel A shows a heatmap representation of relative spliced-in coefficients (*RSICs*) for two exonic regions (E014 [q-value=0, tissue score=2.17] and E016 [q-value=0, tissue score=2.32]) of the gene *SLC25A3* on *subset C* of the *GTEx* data. Panel B shows sashimi plots of the RNA-seq data from the colon and heart samples from individual *11I78*. The highlighted area corresponds to the genomic coordinates of the exons shown in Panel A. These mutually exclusive exons were initially described in an independent study [SR1] that also used RNA-seq data but different bioinformatic methods. As shown in these two panels as well as in figure 1A from the previous study [SR1], this splicing event is regulated differently in heart as compared to other tissues.

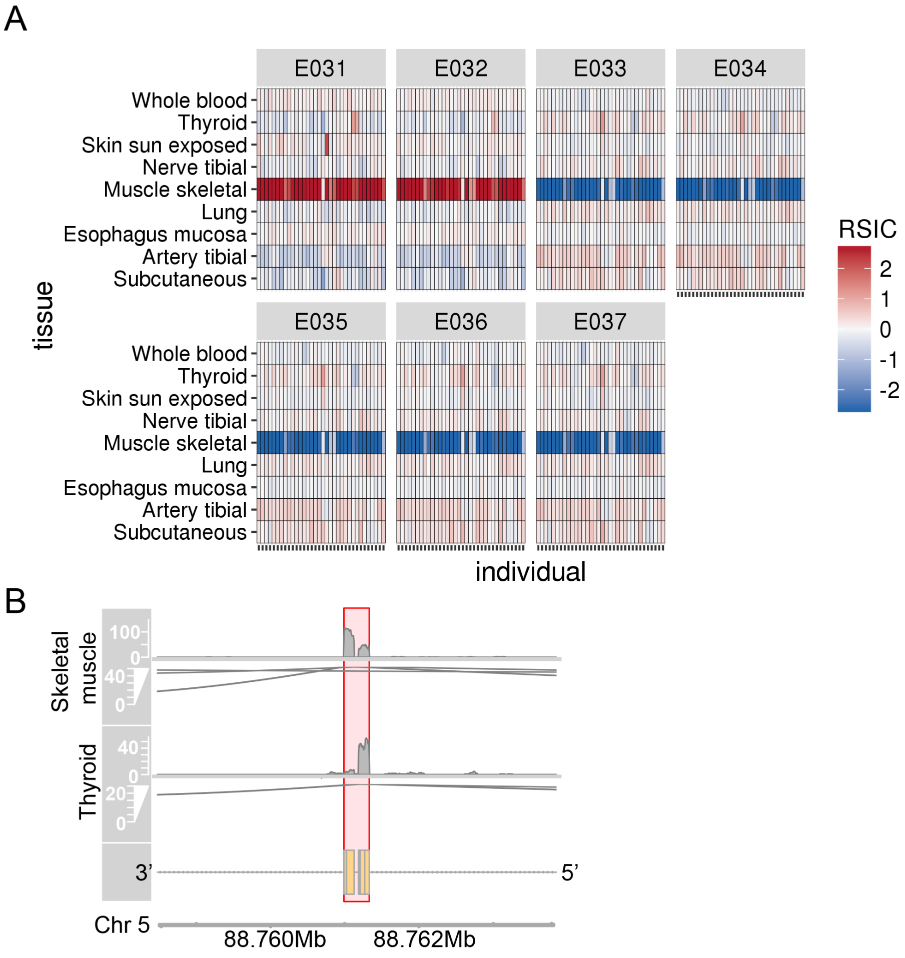

Figure S3: Panel A shows a heatmap representation of relative spliced-in coefficients (*RSICs*) for the exonic regions (E031-E037; all with q-values < 0.1 and tissue scores > 1.5) corresponding to two mutually exclusive alternative exons of the gene *MEF2C* on *subset B* of the *GTEx* data. Panel B shows sashimi plots of the RNA-seq data from the skeletal muscle and thyroid samples from individual *11DXX*. The highlighted area corresponds to the genomic coordinates of the exons shown in Panel A. This splicing event was initially described for mouse tissues in an independent study[SR2] using RT-PCR. This splicing event is regulated differently in skeletal muscle as compared to other tissues. Figure 2C from the original study[SR2] shows the exact same pattern for mouse tissues, which demonstrates that it is a conserved event.

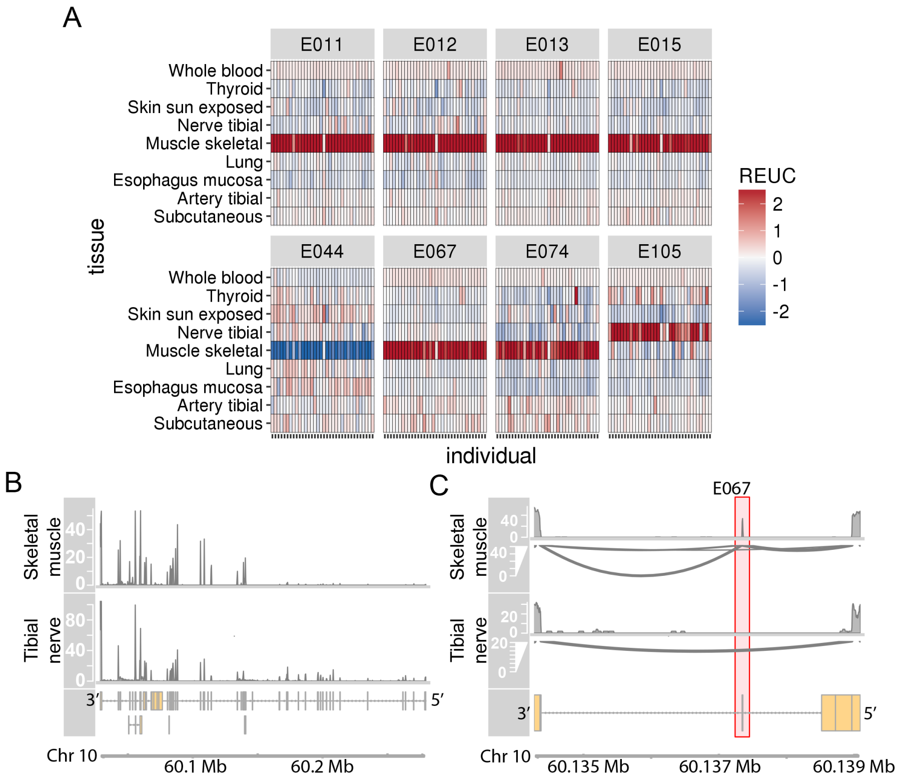

Figure S4: Panel A shows a heatmap representation of relative exon usage coefficients (*REUCs*) for the 8 exonic regions of the gene *ANK3* with the highest tissue scores (all with q-values < 0.1 and tissue scores > 2.19). Panel B shows sashimi plots of the RNA-seq data from the skeletal muscle and tibial nerve samples from individual *11DXX*. Skeletal muscle and tibial nerve initiate transcription from different transcription start sites. Panel C shows sashimi plots (using the same samples as panel B) highlighting an exon-skipping event (exonic region *E067*) that is differentially regulated between skeletal muscle and tibial nerve. These three panels show that *ANK3* transcript isoforms expressed in skeletal muscle are very different to the ones expressed in the rest of the tissues. The expression of skeletal muscle-specific isoforms of *ANK3* has been previously described in rats using northern blots[SR3]. Our analysis of human RNA-seq data substantiates this finding (63 out of the 109 exonic regions of this gene were detected as tissue-dependent), and further indicates that skeletal-muscle undergoes an isoform switch involving both alternative start sites and alternative splicing.

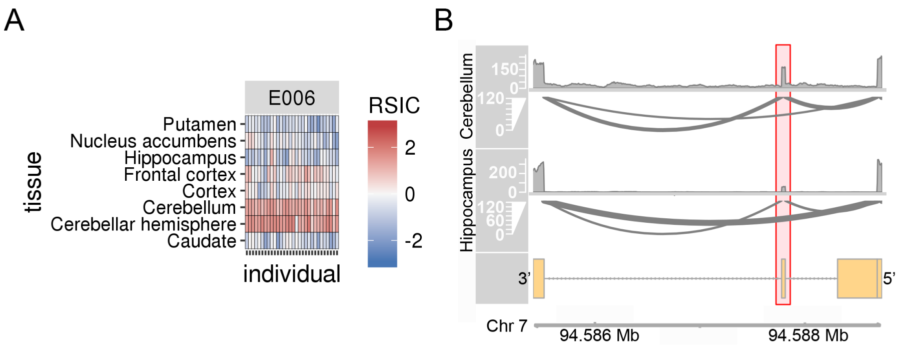

Figure S5: Panel A shows a heatmap representation of the relative spliced-in coefficients (*RSICs*) for the exonic region *E006* (q-values < 0.1 and tissue score > 1.21) of the gene *SGCE*. Panel B shows sashimi plots of the RNA-seq data from the hippocampus and cerebellum samples from individual *12ZZX*. The highlighted region corresponds to the genomic coordinates of the exon *E006*. As shown in these two panels, this cassette event is differentially regulated across brain regions. This pattern of tissue-dependent usage was also observed in another study using qPCR [SR4]: Figure 2 of that study also shows that exon *E006*, labelled there as “11b”, is frequently included in cerebellum as compared to the rest of the brain regions.

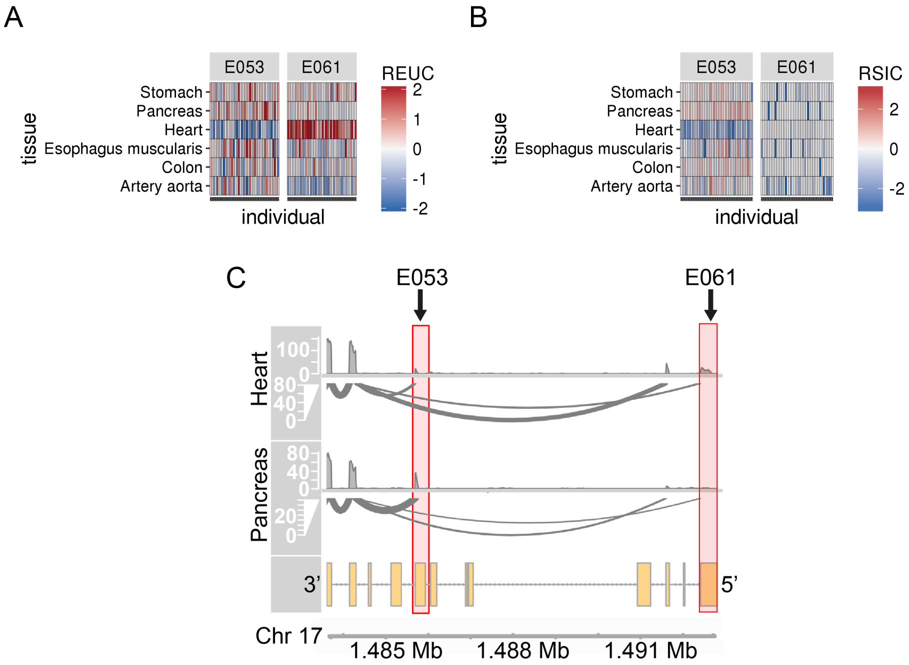

Figure S6: Panel A shows a heatmap representation of the relative exon usage coefficients (*REUCs*) for the exonic regions *E056* (q-values < 0.1 and tissue score = 1.01) and *E061* (q-values < 0.1 and tissue score = 1.32) of the gene *MYO1C*. Panel B shows relative spliced-in coefficients (*RSICs*) for the same exonic regions. Exon *E056* is alternatively spliced, while the usage of *E061* depends on the usage of a transcription start site. Panel C shows sashimi plots of the RNA-seq data from the heart and colon samples from individual *ZF29*. The highlighted regions corresponds to the genomic coordinates of exons *E056* and *E061*. These three panels show that an alternative first exon event is differentially regulated across tissues. While heart uses exon *E061* and splices out exon *E056*, the rest of the tissues use more frequently a TSS in *E056*. This same tissue-dependent event has been previously described in mouse tissues using quantitative real-time PCR[SR5]: in that study, exon *E056* was labelled “Exon -1” (coding for the peptide MRYRA* both in human and mouse) and exon *E061* was labelled as “Exon -2” (coding for the peptide MALQVE* both in human and mouse). Interestingly, while this splicing event is conserved between human and mouse, the patterns of exon inclusion were different between corresponding tissues of the two species (particularly for heart tissue). This observation is consistent with human tissue qPCR data (Maly and Hofmann, 2016, unpublished, personal communication).

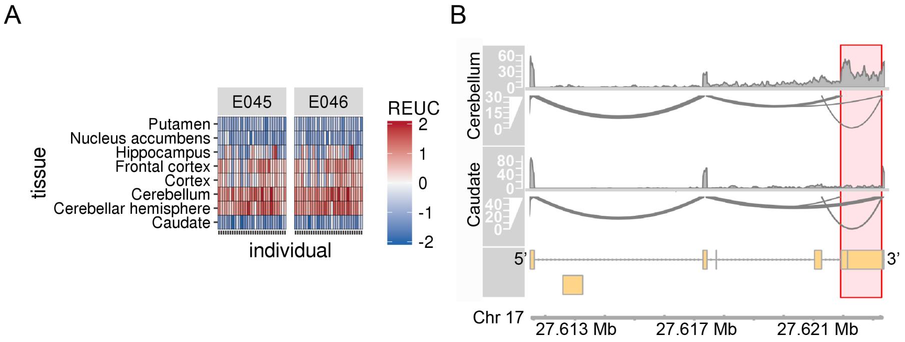

Figure S7: Panel A shows a heatmap representation of the relative exon usage coefficients (*REUCs*) for the exonic regions *E045* (q-values < 0.1 and tissue score = 1.15) and *E046* (q-values < 0.1 and tissue score = 1.18) of the gene *KSR1*. Panel B shows a sashimi plot of the RNA-seq data from the cerebellum and caudate samples from individual *13JVG*. The highlighted regions corresponds to the genomic coordinates of the exons *E045* and *E046*. Our data shows that an alternative splicing event is differentially regulated across brain cell types. The resulting exon inclusion differences are very prominent when comparing cerebellum with caudate samples. This same pattern of tissue-dependent usage has been previously described in mouse using immunoprecipitation assays and immunohistochemical stainings[SR6]. Thus, our data shows that the regulation of this splicing event is conserved between human and mouse.

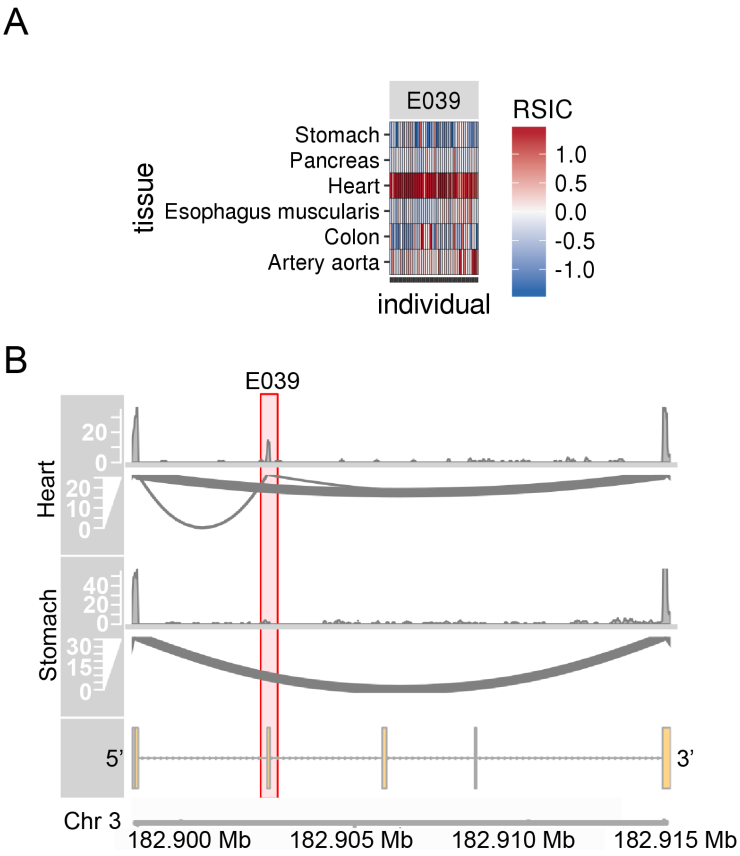

Figure S8: Panel A shows a heatmap representation of the relative spliced-in coefficients (*RSICs*) for the exonic regions *E039* (q-values < 0.1 and tissue score = 1.69) of the gene *ATP11B*. Panel B shows a sashimi plot of the RNA-seq data from the stomach and heart samples from individual *XBED*. The highlighted region corresponds to the genomic coordinates of the exonic region *E039*. Our data shows that an alternative splicing event is regulated in a tissue-specific manner. The inclusion of this exon is elevated in heart tissue as compared to the rest of the tissues. The same pattern of tissue-dependent usage has been previously described using microarray data and RT-PCR[SR7]. Figure 5 of that study also shows elevated exon inclusion of heart tissue as compared stomach tissue.

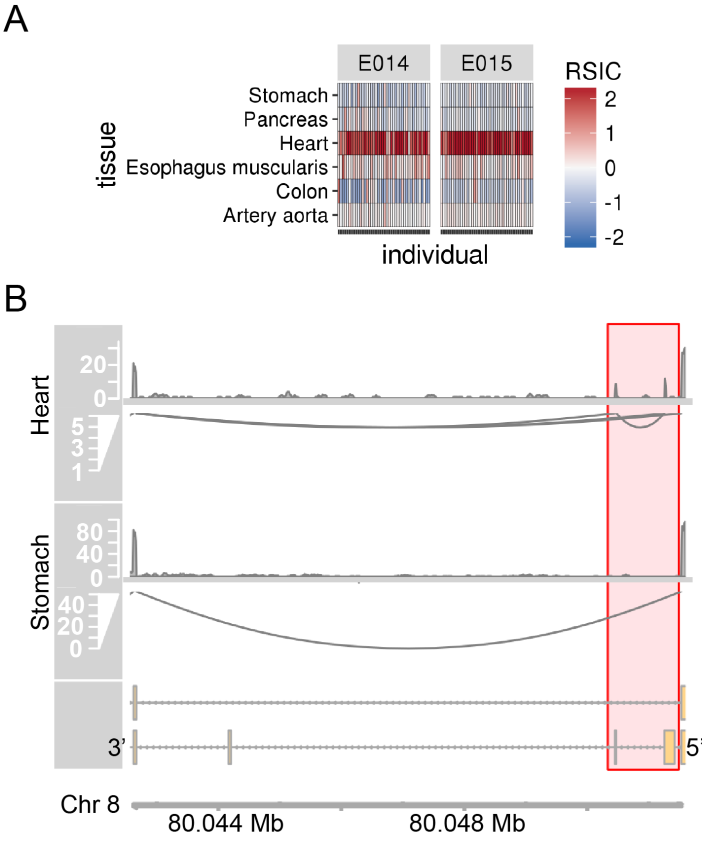

Figure S9: Panel A shows a heatmap representation of the relative spliced-in coefficients (*RSICs*) for two exonic regions, *E014* and *E015* (q-values < 0.1 and tissue scores > 1.7) of the gene *TPD52*. Panel B shows a sashimi plot of the RNA-seq data from the stomach and heart samples from individual *XBED*. The highlighted region corresponds to the genomic coordinates of the exonic regions *E014* and *E015*. Our data shows that these two exonic regions are regulated in a tissue-specific manner. The inclusion of these exons is elevated in heart tissue as compared to the rest of the tissues. The same pattern of tissue-dependent usage has been previously described using microarray data and RT-PCR[SR7]. Figure 5 of that study also shows elevated exon inclusion of heart tissue as compared stomach tissue.

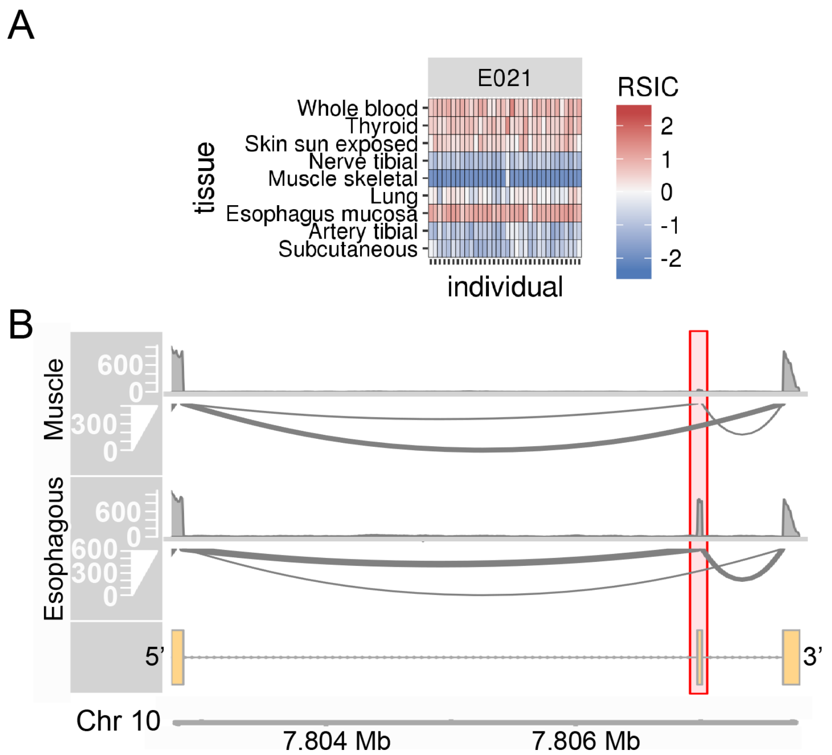

Figure S10: Panel A shows a heatmap representation of the relative spliced-in coefficients (*RSICs*) for exonic regions *E021* (q-values < 0.1 and tissue score $= 2.59$) of the gene *ATP5C1*. Panel B shows a sashimi plot of the RNA-seq data from the esophagous and heart samples from individual *11DXX*. The highlighted region corresponds to the genomic coordinates of the exonic region *E021*. Our data shows strong tissue-dependent inclusion patterns for this exonic region. These differences are particularly pronounced when comparing skeletal muscle with esophagous, thyroid, skin and blood. Using RT-PCR, previous studies have also reported that skeletal muscle excludes this exon from its transcripts[SR8].

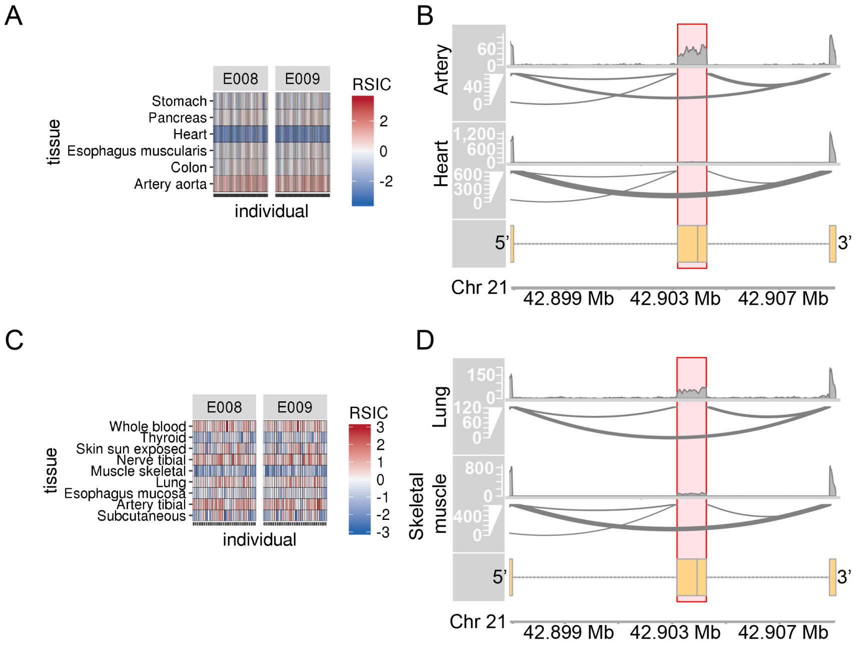

Figure S11: Panels A and C show heatmap representations of the relative spliced-in coefficients (*RSICs*) for exonic regions *E008* and *E009* of the gene *NDUFV3* for two subsets of the *GTEx* data (q-values < 0.1, tissue scores for subset C > 2.02 and tissue scores for subset B > 0.9). Panels B and D show sashimi plots of the RNA-seq data from individuals *11DXZ* (panel B) and *11DXX* (panel D). The highlighted regions correspond to the genomic coordinates of the exonic regions *E008* and *E009*. A previous study[SR9] used mass spectrometry to analyze splicing patterns of this exon in murine and bovine tissues. That study found that while heart and skeletal muscle frequently splice out this exon, brain, liver and lung splice in this exon more frequently. Our analysis of human tissues confirms these findigs, suggesting that this tissue-depenent pattern is also conserved in humans.

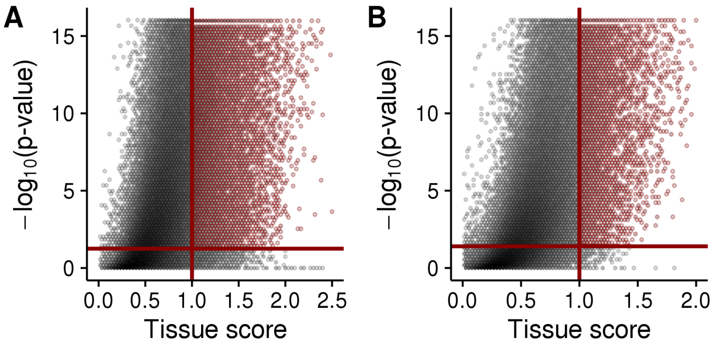

Figure S12: Panels **A** and **B** show data for *subset B* and *subset C* of the GTEx data, respectively. The x-axis shows the tissue dependance score. The y-axis shows the p-value in $-log_{10}$ scale. Each point represent one exonic region. The horizontal lines show the thresholds on both axis used to determine tissue-dependent usage of an exonic region. The red points represent exonic regions above the thresholds (i.e. the exonic regions used in a tissue-dependent manner).

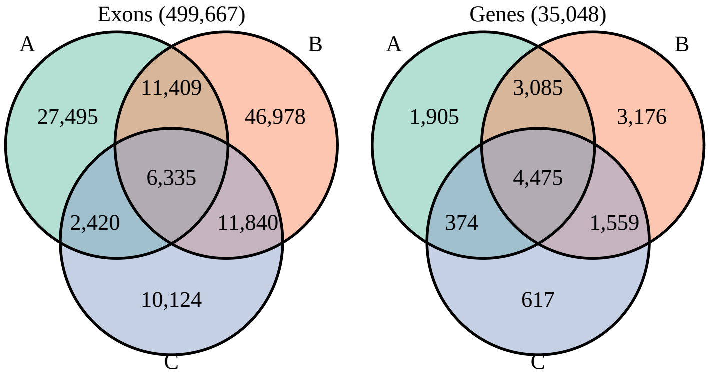

Figure S13: The venn diagram on the left depicts the overlap between exons detected to be used in a tissue-dependent manner in each of the subsets of the GTEx data. The venn diagram on the right depicts the overlap between genes of each subset of the GTEx data where at least one exonic region was detected to be used in a tissue-dependent manner.

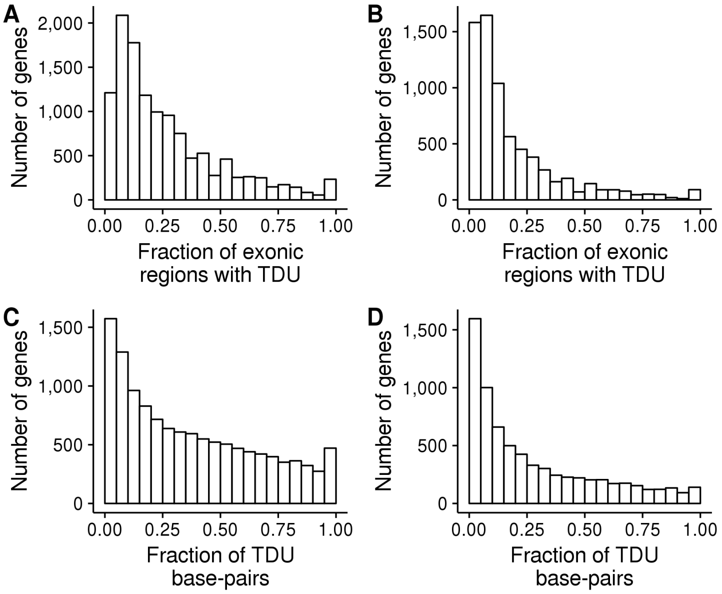

Figure S14: Panels **A** and **C** show data for *subset B*, while panels **C** and **D** show data for *subset C* of the GTEx data. Panels **A** and **B** show histograms of the fraction of exonic regions per gene that are subject to tissue-dependent usage. Panels **C** and **D** show histograms of the fraction of base-pairs of each gene that is subject to tissue-dependent usage.

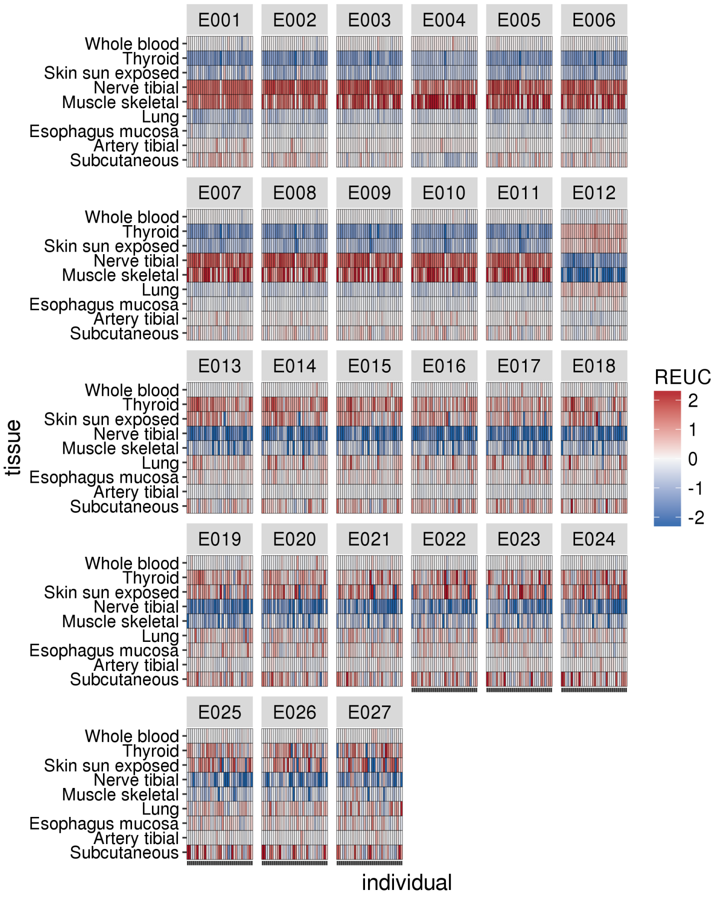

Figure S15: Heatmap representations of the relative exon usage coefficients (*REUCs*) of the exonic regions of the gene *EPB41L4B*. Each panel shows data for one exonic region. Each column of the heatmaps shows data for one individual and each row shows data for one tissue. Colors represent the *REUC* values.

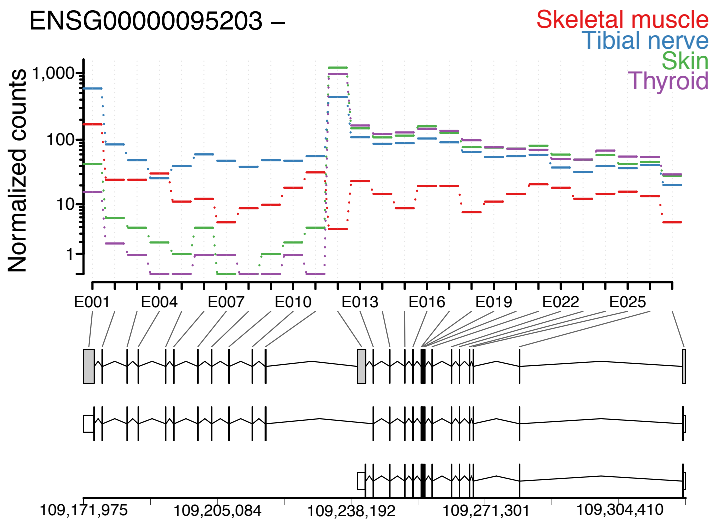

Figure S16: The y-axis of the upper panel represents counts normalized for sequencing depth for each exonic region (x-axis) of the gene *EPB41L4B*. Each line shows data for one tissue of a representative individual (identifier *131XE*). The lower panel shows the annotation of transcript isoforms according to ENSEMBL.

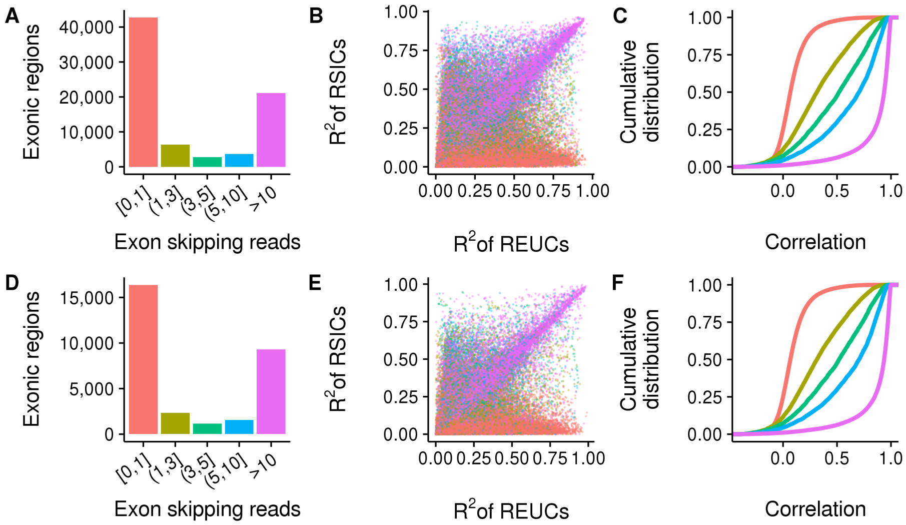

Figure S17: Panels **A**, **B** and **C** show data for *subset B*, while panels **D**, **E** and **D** show data for *subset C* of the GTEx data. **(A, D)** The x-axis shows the number of sequenced fragments supporting exon skipping. The y-axis shows the number of exonic regions with TDU. **(B, E)** Each point represents one exonic region detected to be used in a tissue-dependent manner. The x-axis shows the fraction of total *REUCs* variance that is explained by variance between tissues (R^2). The y-axis shows the R^2 statistic for the RSICs. The colors legends are shown in panels **A** and **D**. **(C, F)** Cumulative distribution (y-axis) of the pair-wise Pearson correlation coefficients between the REUCs and the RSICs (x-axis) of each exonic region with TDU. The data are stratified by the number of sequenced fragments supporting exon skipping (colors legends are shown in panels **A** and **D**).

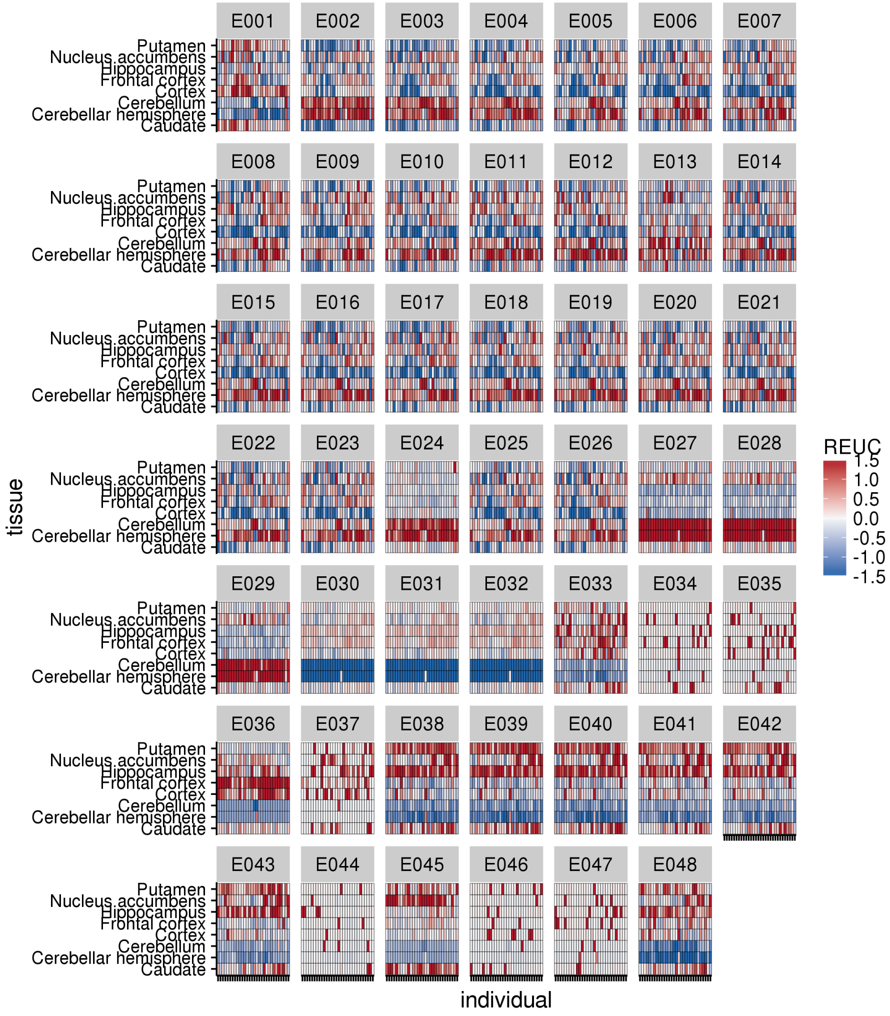

Figure S18: Heatmap representations of the relative exon usage coefficients (*REUCs*) of the exonic regions of the gene *GAS7*. Each panel shows data for one exonic region. Each column of the heatmaps shows data for one individual and each row shows data for one tissue. Colors represent the *REUC* values.

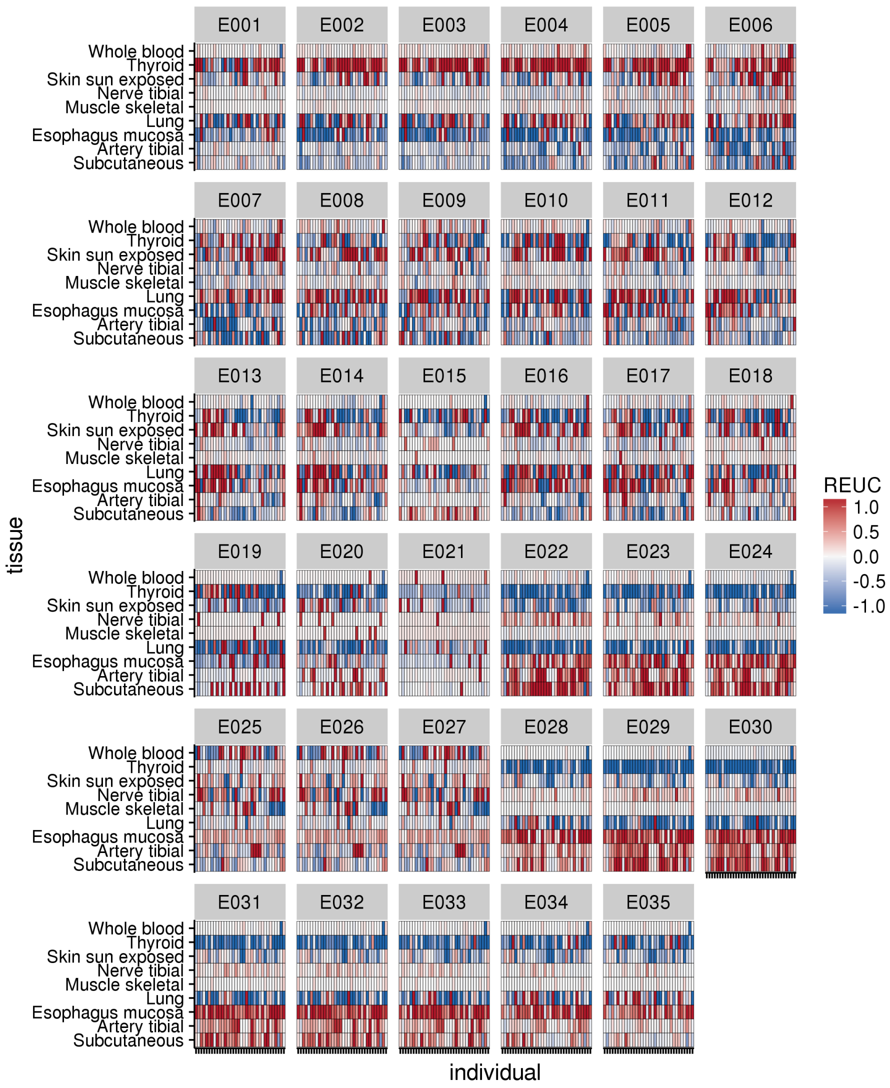

Figure S19: Heatmap representations of the relative exon usage coefficients (*REUCs*) of the exonic regions of the gene *KRT8*. Each panel shows data for one exonic region. Each column of the heatmaps shows data for one individual and each row shows data for one tissue. Colors represent the *REUC* values.

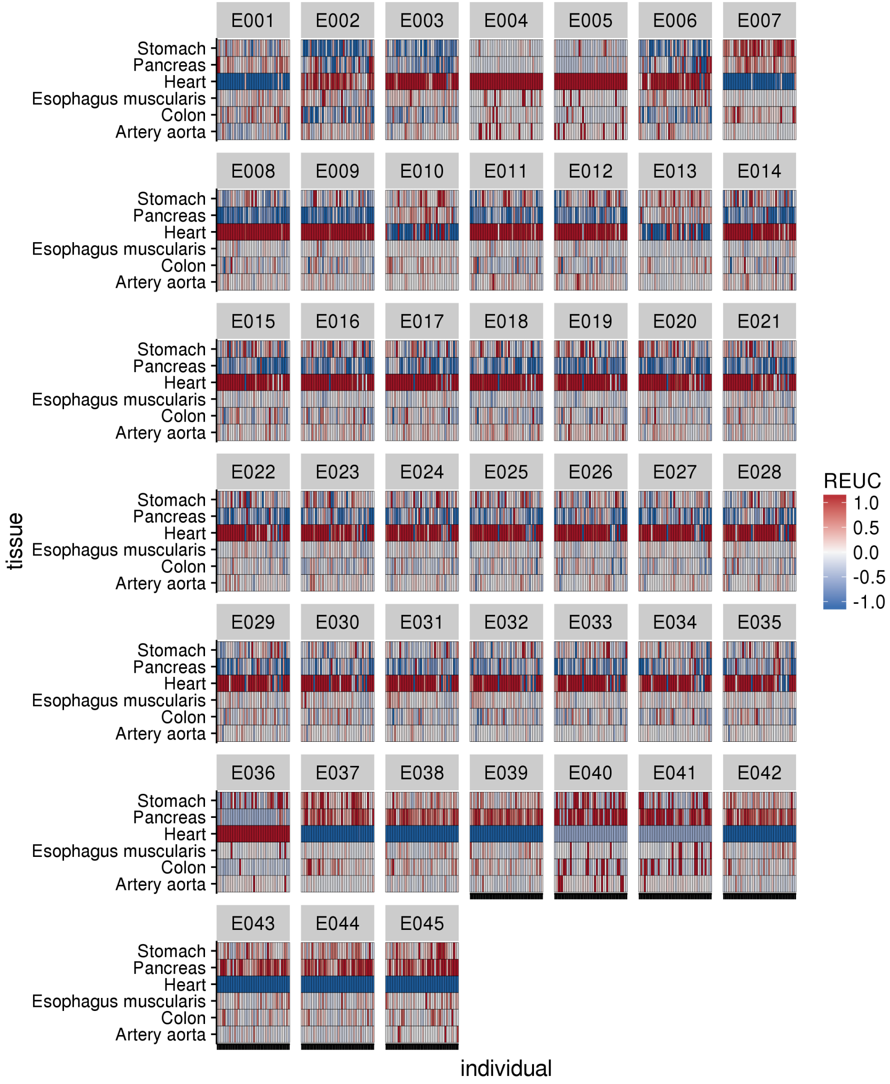

Figure S20: Heatmap representations of the relative exon usage coefficients (*REUCs*) of the exonic regions of the gene *NEBL*. Each panel shows data for one exonic region. Each column of the heatmaps shows data for one individual and each row shows data for one tissue. Colors represent the *REUC* values.

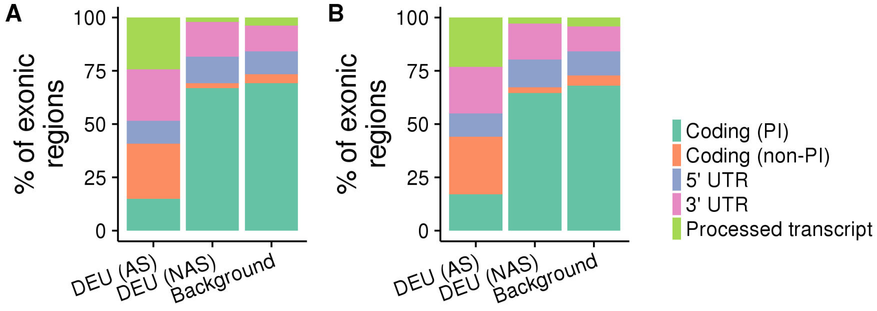

Figure S21: Panel **A** and panel **B** shows data for *subset B* and *subset C* of the *GTEx* data, respectively. The percentage of exonic regions (y-axis) is shown for three sets of exons: (1) exonic regions with TDU due to alternative splicing [DEU (AS)], (2) exonic regions with TDU without strong evidence of alternative splicing [DEU (NAS)] and (3) a background set of exons matched for expression and exon width. Each color represents a different category of exons according to transcript isoforms annotations: exons coding for principal transcript isoforms [Coding (PI)], exons coding for non-principal transcript isoforms [Coding (non-PI)], 5' UTRs, 3' UTRs and exons from non-coding processed transcripts [Processed transcripts].

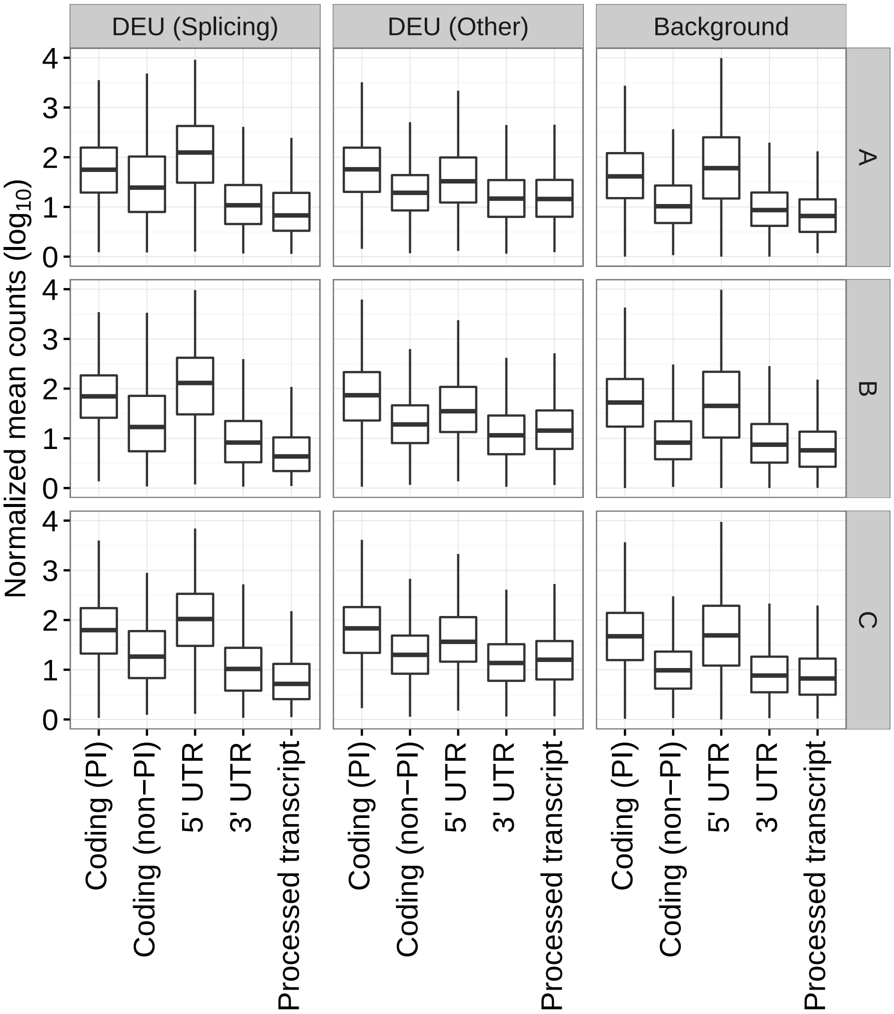

Figure S22: The y-axis shows the mean of normalized counts (in log_{10} scale). Each boxplot shows data for one category of exons according to their transcript isoform annotations (x-axis). The data from each subset of *GTEx* data is plotted in different panels. The data is also plotted in different panels according to their exon classification based on whether (a) the exonic regions are used in a tissue-dependent manner and have evidence of alternative splicing [DEU (Splicing)], (b) the exonic regions are used in a tissue-dependent manner and have no evidence of alternative splicing [DEU (Other)] or (c) the exonic regions belong to the background sets [Background].

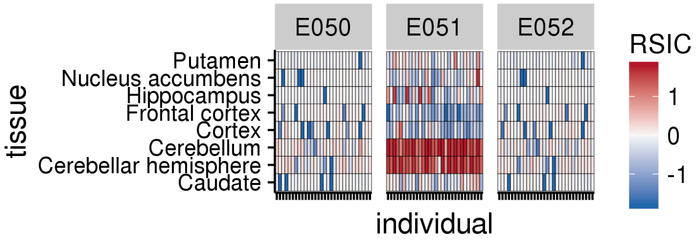

Figure S23: Heatmap representations of the relative spliced-in coefficients (*RSICs*) of three exonic regions of the gene *PKD1*. Each panel shows data for one exonic region. Each column of the heatmaps shows data for one individual and each row shows data for one tissue. Colors represent the *RSIC* values.

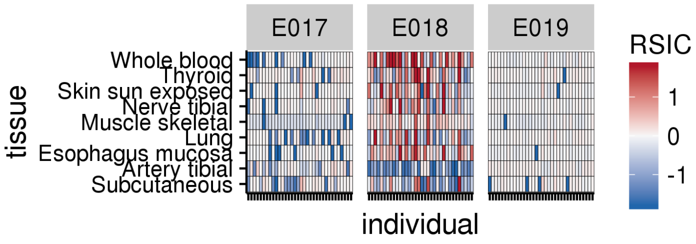

Figure S24: Heatmap representations of the relative spliced-in coefficients (*RSICs*) of three exonic regions of the gene *MAN2B2*. Each panel shows data for one exonic region. Each column of the heatmaps shows data for one individual and each row shows data for one tissue. Colors represent the *RSIC* values.

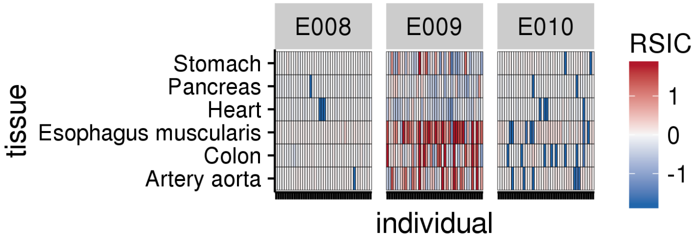

Figure S25: Heatmap representations of the relative spliced-in coefficients (*RSICs*) of three exonic regions of the gene *NISCH*. Each panel shows data for one exonic region. Each column of the heatmaps shows data for one individual and each row shows data for one tissue. Colors represent the *RSIC* values.

2 Supplementary Tables

Study	Profiling method	Number of samples	Total coverage	One-sentence summary of findings related to tissue-specific regulation
Xu et al, 2002 [SR10]	ESTs	4,271 libraries (46 tissues)	2,231,227 ESTs	Ten to 30% of genes showed evidence of tissue-specific splicing. Different tissues exhibit different levels of alternative splicing.
Carnici et al, 2006 [SR11]	CAGE	41 libraries	5,510,369 tags	Around 60% of protein-coding genes use more than a single transcription start site. 92% of genes with at least two TSS are predicted to use distict start codons. Tissue-dependent usage of alternative promoters is frequent.
Clark et al, 2007 [SR7]	Microarrays	16 tissues in triplicates	NA	73% of genes are differentially spliced across tissues. 86% validation rate with RT-PCR.
Wang et al, 2008 [SR1]	RNA-seq	16 tissues (16 samples)	315,043,011 32 bp reads	93% of human genes undergo alternative splicing, 86% with a minor isoform frequency of 15% or more. Most alternative splicing (62%), alternative TSS (52%) and alternative polyadenylation events (65%) are differentially regulated across tissues.
Pan et al, 2009 [SR12]	RNA-seq	6 tissues (6 samples)	136,376,193 32 bp reads	Transcripts from around 95% of human multi-exonic genes undergo alternative splicing (on average, there are 7 alternative splicing events per gene). Most exon-exon junction reads are observed in only one tissue, suggesting that these are tissue-restricted splicing variants.
Barbosa-Morais et al, 2012 [SR13]	RNA-seq	7 tissues across 10 species (171 samples)	8,666,457,099 $\sim$148 bp reads	Splicing has diverged so much through evolution that splicing profiles are more associated to the identity of the species rather than tissue/organ of origin. A minority of alternative splicing events that are conserved across evolution reflect tissue identity.
Merkin et al, 2012 [SR14]	RNA-seq	9 tissues across 5 species (134 samples)	8,670,678,681 $\sim$107 bp reads	Alternative splicing is highly conserved only in a subset of tissues and it is frequently lineage specific. Conserved tissue-specific splicing is enriched among exons that encode for protein phosphorylation sites.
Djebali et al, 2012 [SR15]	RNA-seq	112 samples	16,386,455,980 80 bp reads	Genes express many isoforms simultaneously, with an average of 10 to 12 per gene for one cell-line.
Reyes et al, 2013 [SR16]	RNA-seq	5 tissues across 6 primate species	2,018,132,789 $\sim$ 85 bp reads	Exon usage has more interspecies variability than gene expression levels. Strenght of tissue dependence of an exon is an indicator of the conservation of the regulation.
Ni et al, 2013 [SR17]	PA-seq	13 tissues	107,659,132 $\sim$92 bp reads	Alternative cleavage and polyadenylation is prevalent and sufficient to determine tissue of origin. Different tissues exhibit different patterns of shortening of 3'UTRs.
Lianoglou et al, 2013 [SR18]	3'-seq	14 cell types	535,262,406 $\sim$57 bp reads	While tissue-specific genes tend to have a single cleavage site, ubiquitously expressed genes have multiple cleavage sites. Changes in cleavage sites are not correlated with changes in gene abundance.
Gonzalez-Porta et al, 2013 [SR19]	RNA-seq	19 samples	3,736,859,003 $\sim$ 92 bp reads	Most protein-coding genes express predominantly one isoform, which contributes to the majority of protein products.
Florea et al, 2013 [SR20]	RNA-seq	19 samples	3,736,859,003 $\sim$ 92 bp reads	This study focused on exon skipping events and pairwise comparisons between two tissues. For 523% of alternative splicing events, both isoforms are expressed within the same tissue. 10% to 20% percent of events show different splicing ratios between any two tissues. There are more alternative terminal exons than alternative middle exons.
Forrest et al, 2014 [SR21]	CAGE-seq	1,351 samples	45,189,295,633 $\sim$29 bp reads	Many mammalian promoters consist of multiple closely separated TSS with independent patterns of tissue-specificity. Alternative promoters are often co-expressed across cell-types.
Hestand et al, 2015 [SR22]	RNA-seq	19 samples	3,736,859,003 $\sim$ 92 bp reads	10.3% of exon-exon junctions are expressed in a tissue-restricted manner. 65% of expressed multi-exon genes contain at least one tissue-specific splice junction.
Mele et al, 2015 [SR23]	RNA-seq	1,641 samples	798,362,786,775 $\sim$162 bp reads	Gene expression drives most phenotypic differences across tissues, splicing only plays a complementary role. Splicing might play a bigger role in defining the phenotypic diversity across individuals.

Table S1: Table summarizing studies that analyze transcript isoform differences across human tissues.

	Subset A	Subset B	Subset C
Expressed (> 100 counts)	13,535	13,816	13,205
Expressed and TDU	8,741	10,388	6,124
Percentage	65	75	46

Table S2: Many highly expressed genes are subject to transcript isoform regulation across tissues. Each column shows numbers for one subset of the GTEx data. Row 1: Number of multi-exonic genes with means of normalized sequenced fragments larger than 100. Row 2: Subset of genes from the first row that have evidence of tissue-dependent usage in at least one exonic region. Row 3: Percentage of genes from the first row that have evidence of tissue-dependent usage in at least one exonic region.

	(Foreground) PC	(Foreground) Not PC	(Background) PC	(Background) Not PC	Odds ratio	P-value
A	8,899	940	7,241	2,598	3.40	1.3×10^{-214}
B	10,963	1,332	7,164	2,675	3.07	9.1×10^{-217}
C	6,418	607	7,180	2,659	3.92	5.1×10^{-211}

Table S3: Enrichment of protein coding genes. Each row shows data for one subset of the GTEx data. The first four columns show the number of genes stratified by the categories depicted in the column names (PC - protein coding; foreground - genes with tissue-dependent usage in at least one exonic region; background - genes matched for expression strength and number of exonic regions). The fifth column shows odds ratios and the sixth column shows p-values from the Fisher's exact tests

	# genes with TDU	# genes (< 25% ER TDU)	% genes (< 25% ER TDU)	# genes (< 25% bp TDU)	% genes (< 25% bp TDU)
A	9,839	6,929	70	5,248	53
B	12,295	7,252	59	5,369	44
C	7,025	5,282	75	4,181	60

Table S4: Transcript differences across tissues. Each row shows data for one subset of the GTEx data. The first column shows the number of genes with TDU in at least one exonic region. The second column displays the number of genes with TDU in less than 25% of their exonic regions. The third column shows the percentage of genes from the first row with TDU in less than 25% of their exonic regions. The fourth column displays the number of genes with TDU in less than 25% of their length (excluding introns). The fifth column shows the percentage of genes from the first row with TDU in less than 25% of their length (excluding introns).

Subset	Exon skipping reads (mean)	# of exonic regions	% of exonic regions
A	[0,1]	24,397	51
A	(1,3]	3,628	8
A	(3,5]	1,656	3
A	(5,10]	2,430	5
A	>10	15,548	33
B	[0,1]	42,749	56
B	(1,3]	6,309	8
B	(3,5]	2,721	4
B	(5,10]	3,697	5
B	>10	21,086	28
C	[0,1]	16,385	53
C	(1,3]	2,336	8
C	(3,5]	1,163	4
C	(5,10]	1,553	5
C	>10	9,282	30

Table S5: Evidence of alternative splicing for exons with tissue-dependent usage. For each subset of the GTEx data (first column), the number of exonic regions with TDU (third column) is stratified according to their means of normalized sequenced fragments supporting their splicing out from transcripts (second column). The fourth column shows the percentage of exonic regions in each strata for each subset of data

	A	B	C
# of genes with TDU	9,839	12,295	7,025
# of genes with TDU (fully AS explained)	3,338	2,996	2,492
% of genes with TDU (fully AS explained)	34	24	35

Table S6: Genes with TDEU that could be explained by alternative splicing. Each column shows data for a subset of the GTEx data. The first row shows the number of genes with tissue-dependent usage in at least one exonic region. The second row shows the subset of genes from the first row in which all the exonic regions that are used in a tissue-dependent manner have evidence of being alternatively spliced (normalized mean across samples of exon-skipping reads larger than 1). The third row shows the same quantity as the second row but expressed in percentage of genes.

	Cell-types	# genes	# TDEU	# dTSS	# dTSS and TDEU	% dTSS and TDEU	Odds ratio	P-value
A	Caudate, Cerebellum, Cortex, Hippocampus, Putamen	12,621	9,839	2,402	1,904	79	3.05	1.62×10^{-111}
B	Adipose - Subcutaneous, Lung, Muscle - Skeletal, Skin, Thyroid, Whole Blood	12,548	12,295	6,763	5,427	80	1.83	3.21×10^{-66}
C	Colon, Heart, Pancreas	12,804	7,025	2,778	1,657	60	2.13	1.70×10^{-71}

Table S7: Overlap between genes with differential transcriptional start sites usage and genes with differential exon usage. Each row shows data for one subset of tissues. The first column contains the cell-types available from the FANTOM consortium for each subset of the GTEx data. The second column shows the number of genes that were tested for differential transcription start site (dTSS) usage. The third column shows the number of genes that were tested for dTSS usage that had tissue-dependent exon usage (TDEU). The fourth column shows the number of genes with dTSS usage at a FDR of 10%. The fifth column shows the number of genes with dTSS usage that were also detected to have TDEU. The sixth column shows the percentage of genes with dTSS that were also detected to have TDEU. The seventh column shows odds ratios and the eighth column shows p-values from Fisher's exact tests.

Subset	Exon usage class	Genomic class	# of exons	% of exons
A	DEU (AS)	Coding (PI)	1,870	12.66
A	DEU (AS)	Coding (non-PI)	4,098	27.74
A	DEU (AS)	5' UTR	1,785	12.08
A	DEU (AS)	3' UTR	2,942	19.91
A	DEU (AS)	Processed transcript	4,080	27.61
A	DEU (NAS)	Coding (PI)	16,213	70.13
A	DEU (NAS)	Coding (non-PI)	552	2.39
A	DEU (NAS)	5' UTR	3,354	14.51
A	DEU (NAS)	3' UTR	2,501	10.82
A	DEU (NAS)	Processed transcript	500	2.16
A	Background	Coding (PI)	31,380	69.90
A	Background	Coding (non-PI)	1,812	4.04
A	Background	5' UTR	5,217	11.62
A	Background	3' UTR	5,000	11.14
A	Background	Processed transcript	1,482	3.30
B	DEU (AS)	Coding (PI)	2,959	14.88
B	DEU (AS)	Coding (non-PI)	5,158	25.94
B	DEU (AS)	5' UTR	2,120	10.66
B	DEU (AS)	3' UTR	4,796	24.12
B	DEU (AS)	Processed transcript	4,849	24.39
B	DEU (NAS)	Coding (PI)	27,079	66.92
B	DEU (NAS)	Coding (non-PI)	882	2.18
B	DEU (NAS)	5' UTR	5,095	12.59
B	DEU (NAS)	3' UTR	6,551	16.19
B	DEU (NAS)	Processed transcript	857	2.12
B	Background	Coding (PI)	49,680	69.12
B	Background	Coding (non-PI)	3,021	4.20
B	Background	5' UTR	7,819	10.88
B	Background	3' UTR	8,609	11.98
B	Background	Processed transcript	2,748	3.82
C	DEU (AS)	Coding (PI)	1,515	17.11
C	DEU (AS)	Coding (non-PI)	2,387	26.96
C	DEU (AS)	5' UTR	963	10.88
C	DEU (AS)	3' UTR	1,941	21.92
C	DEU (AS)	Processed transcript	2,049	23.14
C	DEU (NAS)	Coding (PI)	10,019	64.55
C	DEU (NAS)	Coding (non-PI)	426	2.74
C	DEU (NAS)	5' UTR	2,029	13.07
C	DEU (NAS)	3' UTR	2,619	16.87
C	DEU (NAS)	Processed transcript	429	2.76
C	Background	Coding (PI)	19,765	68.09
C	Background	Coding (non-PI)	1,373	4.73
C	Background	5' UTR	3,298	11.36
C	Background	3' UTR	3,389	11.67
C	Background	Processed transcript	1,204	4.15

Table S8: Classification of exonic regions according to their usage across tissues and to transcript isoform annotations. The first column indicates the GTEx subset. The second column indicates exonic region classifications according to whether (a) they were detected to be differentially used and had a mean larger than ten of normalized reads supporting their alternative splicing [DEU (AS)], (b) they were differentially used and had a mean smaller than 1 of normalized read supporting their alternative splicing [DEU (NAS)], or (c) they were part of the background matched for expression strength and width [background]. The third column shows the exonic region classes according to transcript isoform annotations. The fourth column shows the number of exonic regions in each exon class. The fifth column shows, for each usage category on each data subset, the percentage exonic regions in each genomic class.

[SR1] Wang, E. T., Sandberg, R., Luo, S., Khrebtukova, I., Zhang, L., Mayr, C., Kingsmore, S. F., Schroth, G. P., and Burge, C. B. (Nov, 2008) Alternative isoform regulation in human tissue transcriptomes. *Nature,* **456**(7221), 470476.

[SR2] Hakim, N. H. A., Kounishi, T., Alam, A. H. M. K., Tsukahara, T., and Suzuki, H. (Mar, 2010) Alternative splicing of MEF2C promoted by Fox-1 during neural differentiation in P19 cells. *Genes to Cells,* **15**(3), 255267.

[SR3] Hopitzan, A. A., Baines, A. J., Ludosky, M.-A., Recouvreur, M., and Kordeli, E. (Sep, 2005) Ankyrin-G in skeletal muscle: Tissue-specific alternative splicing contributes to the complexity of the sarcolemmal cytoskeleton. *Experimental Cell Research,* **309**(1), 8698.

[SR4] Ritz, K., van Schaik, B. D., Jakobs, M. E., van Kampen, A. H., Aronica, E., Tijssen, M. A., and Baas, F. (Dec, 2010) SGCE isoform characterization and expression in human brain: implications for myoclonusdystonia pathogenesis?. *European Journal of Human Genetics,* **19**(4), 438444.

[SR5] Sielski, N. L., Ihnatovych, I., Hagen, J. J., and Hofmann, W. A. (2014) Tissue specific expression of Myosin IC Isoforms. *BMC Cell Biology,* **15**(1), 8.

[SR6] Muller, J., Cacace, A. M., Lyons, W. E., McGill, C. B., and Morrison, D. K. (Aug, 2000) Identification of B-KSR1, a Novel Brain-Specific Isoform of KSR1 That Functions in Neuronal Signaling. *Molecular and Cellular Biology,* **20**(15), 55295539.

[SR7] Clark, T. A., Schweitzer, A. C., Chen, T. X., Staples, M. K., Lu, G., Wang, H., Williams, A., and Blume, J. E. (2007) Discovery of tissue-specific exons using comprehensive human exon microarrays. *Genome Biology,* **8**(4), R64.

[SR8] Hayakawa, M., Sakashita, E., Ueno, E., Tominaga, S.-i., Hamamoto, T., Kagawa, Y., and Endo, H. (Dec, 2001) Muscle-specific Exonic Splicing Silencer for Exon Exclusion in Human ATP Synthase gamma-subunit pre-mRNA. *Journal of Biological Chemistry,* **277**(9), 69746984.

[SR9] Guerrero-Castillo, S., Cabrera-Orefice, A., Huynen, M. A., and Arnold, S. (Mar, 2017) Identification and evolutionary analysis of tissue-specific isoforms of mitochondrial complex I subunit NDUFV3. *Biochimica et Biophysica Acta (BBA) - Bioenergetics,* **1858**(3), 208217.

[SR10] Xu, Q., Modrek, B., and Lee, C. (Sep, 2002) Genome-wide detection of tissue-specific alternative splicing in the human transcriptome. *Nucleic Acids Research,* **30**(17), 3754–3766.

[SR11] Carninci, P., Sandelin, A., Lenhard, B., Katayama, S., Shimokawa, K., Ponjavic, J., Semple, C. A. M., Taylor, M. S., Engstrm, P. G., Frith, M. C., and et al. (Apr, 2006) Genome-wide analysis of mammalian promoter architecture and evolution. *Nature Genetics,* **38**(6), 626635.

[SR12] Pan, Q., Shai, O., Lee, L. J., Frey, B. J., and Blencowe, B. J. (Nov, 2008) Deep surveying of alternative splicing complexity in the human transcriptome by high-throughput sequencing. *Nature Genetics,* **40**(12), 14131415.

[SR13] Barbosa-Morais, N. L., Irimia, M., Pan, Q., Xiong, H. Y., Gueroussov, S., Lee, L. J., Slobodeniuc, V., Kutter, C., Watt, S., Colak, R., and et al. (Dec, 2012) The Evolutionary Landscape of Alternative Splicing in Vertebrate Species. *Science,* **338**(6114), 15871593.

[SR14] Merkin, J., Russell, C., Chen, P., and Burge, C. B. (Dec, 2012) Evolutionary Dynamics of Gene and Isoform Regulation in Mammalian Tissues. *Science,* **338**(6114), 15931599.

[SR15] Djebali, S., Davis, C. A., Merkel, A., Dobin, A., Lassmann, T., Mortazavi, A., Tanzer, A., Lagarde, J., Lin, W., Schlesinger, F., and et al. (Sep, 2012) Landscape of transcription in human cells. *Nature,* **489**(7414), 101108.

[SR16] Reyes, A., Anders, S., Weatheritt, R. J., Gibson, T. J., Steinmetz, L. M., and Huber, W. (Sep, 2013) Drift and conservation of differential exon usage across tissues in primate species. *Proceedings of the National Academy of Sciences,* **110**(38), 1537715382.

[SR17] Ni, T., Yang, Y., Hafez, D., Yang, W., Kiesewetter, K., Wakabayashi, Y., Ohler, U., Peng, W., and Zhu, J. (2013) Distinct polyadenylation landscapes of diverse human tissues revealed by a modified PA-seq strategy. *BMC Genomics,* **14**(1), 615.

[SR18] Lianoglou, S., Garg, V., Yang, J. L., Leslie, C. S., and Mayr, C. (Oct, 2013) Ubiquitously transcribed genes use alternative polyadenylation to achieve tissue-specific expression. *Genes & Development,* **27**(21), 23802396.

[SR19] Gonzalez-Porta, M., Frankish, A., Rung, J., Harrow, J., and Brazma, A. (2013) Transcriptome analysis of human tissues and cell lines reveals one dominant transcript per gene. *Genome Biology,* **14**(7), R70.

[SR20] Florea, L., Song, L., and Salzberg, S. L. (Sep, 2013) Thousands of exon skipping events differentiate among splicing patterns in sixteen human tissues. *F1000Research,*.

[SR21] Forrest, A. R. R., Kawaji, H., Rehli, M., Kenneth Baillie, J., de Hoon, M. J. L., Haberle, V., Lassmann, T., Kulakovskiy, I. V., Lizio, M., Itoh, M., and et al. (Mar, 2014) A promoter-level mammalian expression atlas. *Nature,* **507**(7493), 462470.

[SR22] Hestand, M. S., Zeng, Z., Coleman, S. J., Liu, J., and MacLeod, J. N. (Dec, 2015) Tissue Restricted Splice Junctions Originate Not Only from Tissue-Specific Gene Loci, but Gene Loci with a Broad Pattern of Expression. *PLOS ONE,* **10**(12), e0144302.

[SR23] Mele, M., Ferreira, P. G., Reverter, F., DeLuca, D. S., Monlong, J., Sammeth, M., Young, T. R., Goldmann, J. M., Pervouchine, D. D., Sullivan, T. J., and et al. (May, 2015) The human transcriptome across tissues and individuals. *Science,* **348**(6235), 660665.
